# MHCII-peptide presentation: an assessment of the state-of-the-art prediction methods

**DOI:** 10.3389/fimmu.2024.1293706

**Published:** 2024-03-12

**Authors:** Yaqing Yang, Zhonghui Wei, Gabriel Cia, Xixi Song, Fabrizio Pucci, Marianne Rooman, Fuzhong Xue, Qingzhen Hou

**Affiliations:** ^1^ Department of Biostatistics, School of Public Health, Cheeloo College of Medicine, Shandong University, Jinan, China; ^2^ National Institute of Health Data Science of China, Shandong University, Jinan, China; ^3^ Computational Biology and Bioinformatics, Université Libre de Bruxelles, Brussels, Belgium; ^4^ Interuniversity Institute of Bioinformatics in Brussels, Brussels, Belgium

**Keywords:** MHCII, peptide binding prediction, immunology, bioinformatics, webserver, machine learning

## Abstract

Major histocompatibility complex Class II (MHCII) proteins initiate and regulate immune responses by presentation of antigenic peptides to CD4+ T-cells and self-restriction. The interactions between MHCII and peptides determine the specificity of the immune response and are crucial in immunotherapy and cancer vaccine design. With the ever-increasing amount of MHCII-peptide binding data available, many computational approaches have been developed for MHCII-peptide interaction prediction over the last decade. There is thus an urgent need to provide an up-to-date overview and assessment of these newly developed computational methods. To benchmark the prediction performance of these methods, we constructed an independent dataset containing binding and non-binding peptides to 20 human MHCII protein allotypes from the Immune Epitope Database, covering DP, DR and DQ alleles. After collecting 11 known predictors up to January 2022, we evaluated those available through a webserver or standalone packages on this independent dataset. The benchmarking results show that MixMHC2pred and NetMHCIIpan-4.1 achieve the best performance among all predictors. In general, newly developed methods perform better than older ones due to the rapid expansion of data on which they are trained and the development of deep learning algorithms. Our manuscript not only draws a full picture of the state-of-art of MHCII-peptide binding prediction, but also guides researchers in the choice among the different predictors. More importantly, it will inspire biomedical researchers in both academia and industry for the future developments in this field.

## 1 Introduction

MHCII molecules are transmembrane glycoprotein heterodimers, which capture exogenous antigenic peptides through their peptide binding region and present them to CD4+ T cells to initiate the immune response (1–3). The binding between MHCII proteins and antigenic peptides is the key step for T-cells to recognize non-self or tumor-associated antigens and thus to initiate humoral and cellular immunity (4–7). The peptide binding region of MHCII is situated at the interface between the two chains *α* and *β* of MHCII, unlike MHCI proteins where it is found in the *α* chain (8, 9). The binding groove is open at both ends which allows binding peptides of various lengths. Moreover, the high polymorphism of MHCII genes resulting from a variety of different alleles at each locus (i.e., HLA-DR, -DP and -DQ) make MHCII-peptide binding prediction even more complicated (2, 9, 10). Other factors such as the flanking sequences of the peptides and the diversity of peptide binding cores also affect the MHC-peptide binding prediction (11, 12). Thus, the binding specificity of MHCII alleles is really challenging to predict.

Great efforts have been devoted to the experimental identification of peptides binding to MHC molecules (13, 14) through a variety of approaches such as competitive binding assays (15, 16) and ELISPOT assays (17), as well as mass spectrometry methods (18–20). These approaches usually evaluate the MHC-peptide interactions experimentally by quantifying their binding affinity and/or immunogenicity (14, 21). Moreover, considering the importance of MHCII proteins in selecting peptides for antigen presentation and the coordination of immune responses, high-throughput experiments have also been developed for identifying the correct MHC-peptide interactions at peptidomic or genomic scale (19, 22). However, despite continuous improvements over the last decade, the expensive and time-consuming experimental techniques for large-scale determination of MHC-binding peptides are not yet able to provide comprehensive coverage of such interactions (23).

In parallel, many computational methods have been developed to facilitate MHCII-peptide identification. They are becoming increasingly important in large-scale scanning of neoantigens, tumor vaccine development and drug design (24–29). There are in general three types of computational approaches to predict the likelihood of MHC-peptide binding: scoring functions, machine-learning based methods and consensus approaches (30–33). In recent years, predictors based on extensive MHC ligand and binding data (34), such as NetMHCIIpan-4.1 (23) and MixMHC2pred (35), have reached very high prediction accuracy with area under the receiver operating characteristic (ROC) curve (AUC) above 0.9 (23, 35).

It is an interesting task to review the MHCII prediction approaches and compare their performance, since many novel methods have recently been developed. Although there are several nice reviews and assessments on MHCI-peptide binding (36), the most recent review about MHCII-peptide binding prediction dates back several years (37–40). There is thus an urgent need for a comprehensive review and a complete assessment of these predictors. In this paper, we first provide an overview of 11 peptide-MHCII binding predictors, including a description of their algorithms, features, availability and performance. More importantly, we constructed a new independent dataset consisting of positive (binding) and negative (non-binding) peptides for HLA-DP, -DQ and -DR alleles by collecting the most recent MHCII-peptide binding data from the Immune Epitope Database (IEDB) (34) and filtering out the sequences included in the training sets of the evaluated predictors. The performance of each method on our independent benchmark dataset shows that different predictors are best at predicting different alleles, which can guide the use of certain predictors for specific alleles. This review and assessment of the state-of-the-art MHCII-peptide binding methods will be useful for researchers in the field and contribute to boost the development of personalized vaccine and immunotherapy.

## 2 Materials and methods

### 2.1 Construction of validation data set

The predictive performance of data-driven computational models greatly depends on the amount and quality of the validation datasets. In this work, we thus carefully curated a new benchmark dataset by a thorough process to avoid possible overlap between our benchmark dataset and the training datasets of the evaluated methods.

We started by downloading peptide sequences from IEDB (34), which is the largest public resource for MHC ligands and T-cell epitopes. In order to avoid redundancies, we selected IEDB data deposited after 2020, named *Dset_ini_*, since most of the predictors used for their training the NetMHCIIpan3.2 (41) dataset that is basically constructed from IEDB before 2020. Next, we removed the peptides that were 100% identical to those appearing in IEDB before 2020 (*Dset_b_*_2020_). From the resulting dataset we retained the top 20 HLA allotypes with the highest amount of data. This led to the final positive dataset *Dset_pos_* which contains in total 67,061 peptides that vary in length between 11 and 19 residues, and bind with the 20 different HLA allotypes (9 DR, 1 DQ and 10 DP allotypes).

**Figure 1:**
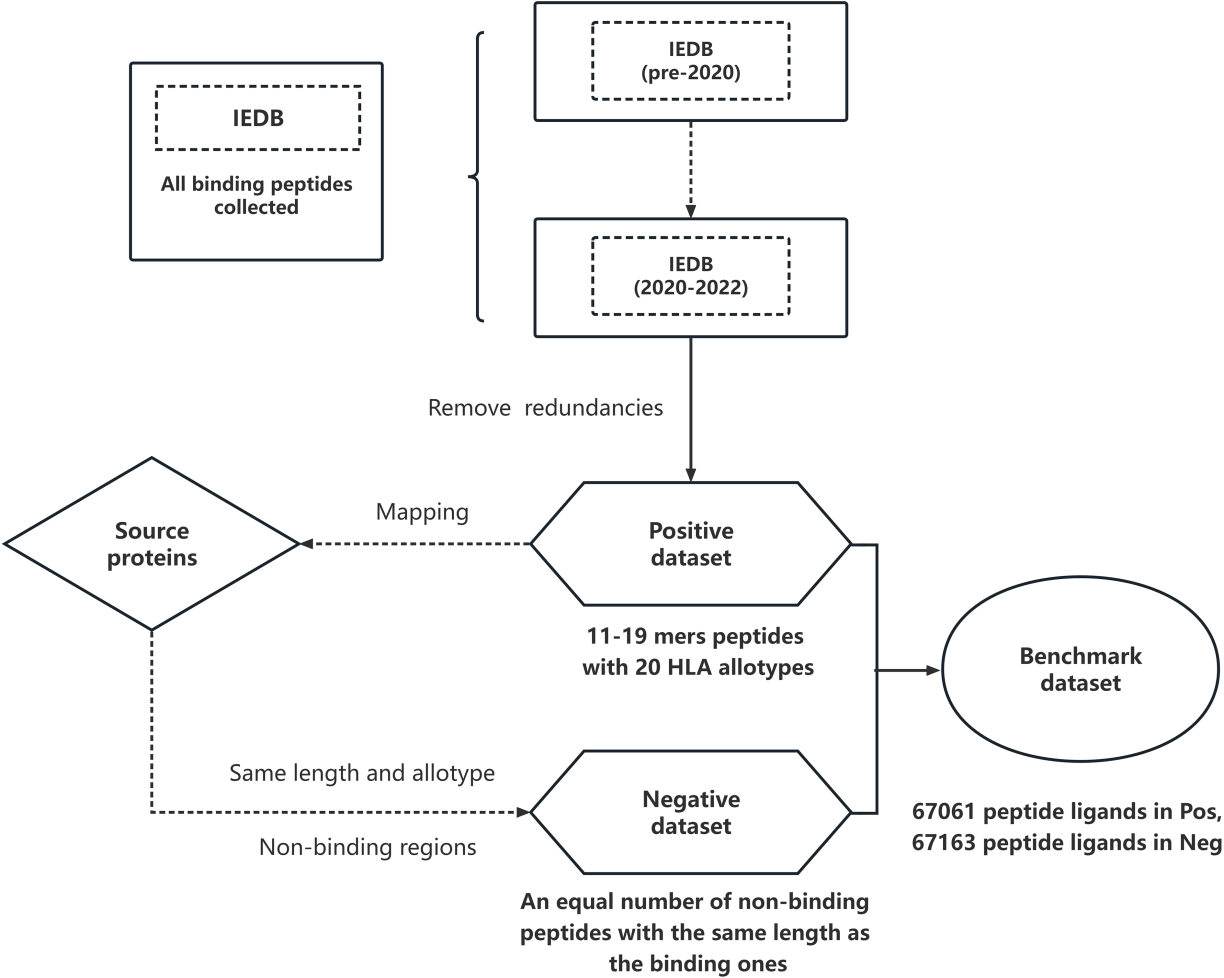
Overall workflow of the construction of the benchmark dataset: we first collected all peptides from IEDB and selected data after 2020 (2020-2022) as our starting dataset *Dset_ini_*. We removed the overlap between *Dset_ini_* and the IEDB dataset entries before 2020 to make the positive *Dset_pos_* dataset containing 67061 binding peptides of 11 to 19 amino acids; they span 20 allotypes. We used the non-bound regions of the host proteins to generate non-binding peptides of the same length as the binding ones. After that, we removed the non-binding peptides that appear in IEDB. The remaining peptides constitute the negative *Dset_neg_* dataset of 67163 non-binding peptides. The union of *Dset_pos_* and *Dset_neg_* is the final benchmark dataset *Dset_bench_*.

To avoid bias and imbalance issues when comparing the methods, we also generated an equal number of MHCII non-binding peptides. After obtaining the full sequence of the antigen proteins for each binding peptide in the positive dataset, we mapped all binding peptides to each antigen. For each group of peptides of a given length interacting with the same MHCII protein, we randomly generated an equal number of peptides from the non-binding sequence regions of the antigen proteins. During the construction of the negative dataset, we filtered out the peptides that occur in the IEDB as well as in the training sets of other predictors to make the dataset as clean as possible. In this way we generated a negative dataset that contains 67,163 peptides in total, with a length ranging from 11 to 19 residues. The final benchmark dataset *Dset_bench_* includes both positive and negative binding peptides. Its construction is schematically shown in **Figure 1**.

The *Dset_bench_* and *Dset_ini_* both contain HLA-DP, HLA-DR and HLA-DQ alleles but with different proportions. Indeed, the DP, DR and DQ fractions in *Dset_ini_* are equal to 36% (93,777), 57% (150,734), and 7% (15,929), respectively, whereas they are equal to 65% (43,463), 31% (21,103) and 4% (2,495) in *Dset_bench_*. DR data thus constitutes the majority of *Dset_ini_*, as in the full IEDB. The removal of redundant entries in *Dset_ini_* leads to proportionally more DPs and less DRs in our benchmark *Dset_bench_*. In order to evaluate the prediction methods on the different alleles separately, we splitted *Dset_bench_* into *DRset_bench_*, *DPset_bench_* and *DQset_bench_*, with each subset containing only DR, DP, and DQ allele peptides, respectively.

It is important to note that there might be some ‘negative’ peptides that are incorrectly classified, as we cannot totally exclude that they still bind to MHCII proteins. However, this is the maximum precision that we can achieve with the available data and, moreover, the comparison between the predictors remains fair even if there are some misclassifications. Also note that the vast majority of peptides in the different datasets were identified using mass spectrometry experiments, which are less accurate than standard biochemical assays. Indeed, mass spectrometry-based peptide identification typically suffers from about 1% false discovery rate (42). However, it can be argued that the fraction of false peptides in *Dset_bench_* is higher. Indeed, peptides found twice in two independent experiments are more likely to be correct. Therefore, assuming the worst case scenario, filtering of the *Dset_ini_* dataset for redundancy using *Dset_b_*_2020_ removes exclusively true HLA binding peptides. In this hypothesis, the fraction of false peptides for DP, DR and DQ alleles in *Dset_bench_* is of about 2%, 7% and 6%, respectively. Despite these slightly higher experimental errors, their potential impact on the benchmark results remain limited, especially for the ranking of the different predictors for each allele.

Our curation process led to an independent, balanced and unbiased dataset, which is important to rigorously benchmark prediction methods. This benchmark dataset constitutes a new reference dataset and is available in **Supplementary Data S1**.

### 2.2 Performance evaluation strategy

We tested all the predictors using the independent dataset described above. The prediction scores given by the tested predictors, used to rank the MHCII-peptide interactions, varies between IC50 binding values in nM, eluted ligand likelihood prediction scores (EL-score), and binding affinity prediction scores (BA-score). When the predictors output multiple scores, we selected the score that gives the best prediction performance.

The evaluation of performance is quantified as follows:

True positives (TP) are correct predictions of HLA-peptide interactions; false positives (FP) are peptides that are incorrectly predicted to bind with HLA, true negatives (TN) are non-binding peptides that were correctly recognized; and false negatives (FN) are interacting peptides which were wrongly predicted as non-interacting. We chose the AUC as a measure of predictors performance. The plotting of the ROC curve and the calculation of AUC were all carried out with the ROCR package for R (43).

Not all the methods set a cut-off for the binary, binding or non-binding, prediction. We also evaluated the F1 and BACC (Balanced accuracy) to search for the best cut-off for each method. The equations of F1 and BACC are defined as follows:


Precision = TP/(TP+FP)

Sensitivity = TP/(TP+FN)

Specificity =TN/(TN+FP)

BACC = (Sensitivity+Specificity)/2

F1 = 2×Precision×Sensitivity/(Precision+Sensitivity)

### 2.3 Peptide alignment

To perform the conservation analysis in our datasets, we collected the peptides binding with the same allele ignoring their length. MAFFT (for multiple alignment using fast Fourier transform) (44) was used to align the binding peptides for each of the 20 HLAII proteins. The open gap penalty was set to 4 to avoid adding gaps in the peptides. After aligning the peptides binding to a given allele, we counted the number of gaps at each position of the alignment and selected only the contiguous positions with less than 10 percent gaps in the center regions of the MSA (Multiple Sequence Alignment). From this MSA, we selected the peptides that are aligned without gaps in the 9-residue regions as our starting alignments. We then realigned all remaining peptides that have gaps to the previous alignment using the MAFFT program. The final alignment included the 9-residue binding cores without gaps. A few sequences were dropped since they still contained gaps after two alignment iteration rounds.

### 2.4 Existing peptide-binding prediction tools

**Figure 2:**
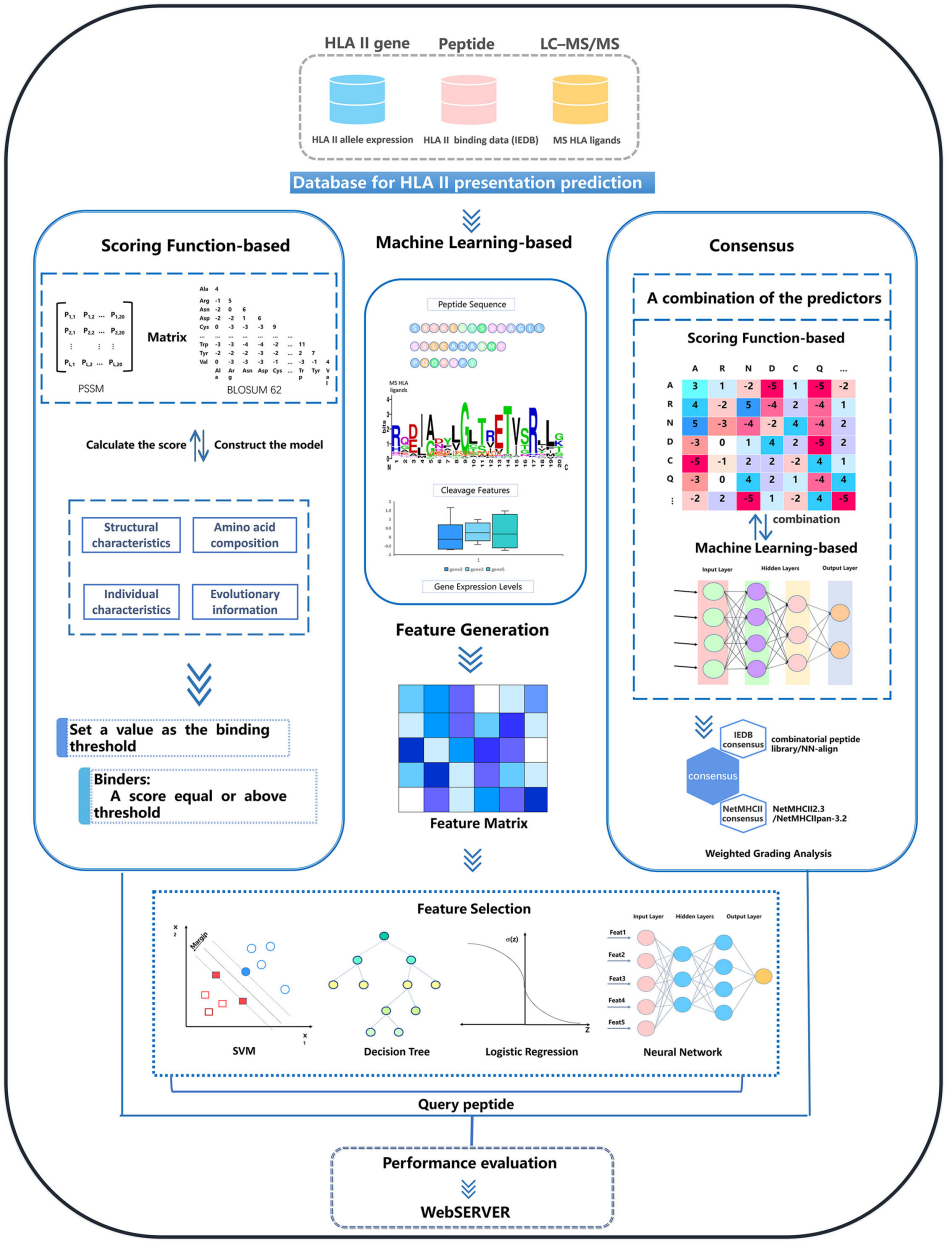
Overview of the computational approaches for MHCII-peptide interaction prediction. There are three types of predictors: scoring functions, machine-learning based tools, and consensus approaches. For each type of method, there are generally five steps to build a reliable predictor: data acquisition and preprocessing, feature generation and selection, model construction and optimization, performance evaluation, and the construction of a web server or independent software.

The currently available tools are divided into three main categories based on the algorithms they used: scoring functions, machine learning and consensus methods. The general workflows of these three types of methods are schematically illustrated in **Figure 2**. **Table 1** shows a summary of existing MHCII-peptide binding predictors. In the following, we briefly describe the 11 selected predictors, emphasizing their main characteristics in terms of algorithms, features, training datasets and availability (see **Table 1**).

**Table 1:** Summary of MHCII-peptide binding prediction tools.

Category	Method name	Algorithms	Year	Availability	nput data	Peptide ength	Pan-specific	Threshold	Features	Last update	URL	HLA type
**Machine-learning based**	MARIA	multimodal deep neural network	2019	webserver	HLA type/peptide sequence/gene symbol	8∼26 mer	YES	95% percentile score	Sequence-based features	2022	https://maria.stanford.edu/	DR/DQ
	MHCnuggets	LSTM	2019	github codes	HLA type/peptide sequence	30 mer	NO	500 nM	Sequence-based features	2021	https://github.com/KarchinLab/mhcnuggets	DR/DQ/DP
	MixMHC2pred	MoDec	2019	webserver/github codes	HLA type/peptide sequence	8∼25 mer	YES	top 20%	Sequence-based features (motifs/length)	2020	http://mixmhc2pred.gfellerlab.org/	DR/DQ/DP(12-21 mer)
	BERTMHC	Transformer neural network model MIL	2021	webserver	HLA type/ HLA peptide sequence	13∼21 mer	YES	500 nM	Sequence-based features	2021	https://github.com/s6juncheng/BERTMHC	DR/DQ/DP
	NetMHCIIpan-4.1	NNAlign_MA/ANN	2020	webserver	HLA Type/ HLA peptide sequences/ Context encoding	9 mer	YES	%Rank EL (5%)	Sequence-based & binary features	2020	https://services.healthtech.dtu.dk/service.php?NetMHCIIpan-4.1	DR/DQ/DP/Mouse(H-2)
	NetMHCIIpan-4.0	NNAlign_MA/ANN	2020	webserver	HLA Type/ HLA peptide sequences/ Context encoding	9 mer	YES	%Rank EL (10%)	Sequence-based & binary features	2020	https://services.healthtech.dtu.dk/service.php?NetMHCIIpan-4.0	DR/DQ/DP/Mouse(H-2)
	NetMHCII-2.3	NNAlign/ ANN	2017	webserver	HLA type/ HLA peptide sequence	9∼25 mer	NO	500nM	Sequence-based features	2017	http://www.cbs.dtu.dk/services/NetMHCII-2.3/	DR/DQ/DP/Mouse(H-2)
	NetMHCIIpan-3.2	NNAlign/ ANN	2017	webserver	HLA type/ HLA peptide sequence	9∼25 mer	YES	500nM	Sequence-based features	2017	https://services.healthtech.dtu.dk/service.php?NetMHCIIpan-3.2	DR/DQ/DP/Mouse(H-2)
**Scoring function based**	NetMHCII	SMM-align/ Gibbs sampler weight matrices	2007	github codes	HLA type/ HLA peptide sequence	12∼18 mer	NO	500nM	NA	2007	http://tools.immuneepitope.org/mhcii/	DR/DQ/DP/Mouse(H-2)
	MHCII3D	Statistical potential	2021	webserver	HLA type/ HLA peptide sequence	9 mer	NO	500nM	NA	2018	https://pbwww.services.came.sbg.ac.at/?page_id=762	DR
**Consensus**	IEDB consensus	Combination of rank predicted from several methods	2008/ 2010	webserver	HLA peptide sequence/peptide gene	12∼18 mer	NO	1000nM	NA	2019	http:/tools.immuneepitope.org/mhcii/	DR/DQ/DP/Mouse(H-2)

#### 2.4.1 Scoring functions

Scoring functions are widely used to assess MHCII-peptide binding properties (36, 45). Most of these methods include only sequence information. They are based on position-specific scoring matrices (PSSM) which represent the frequency of each amino acid at each position in the ensemble of peptides binding to a given MHCII allele. Only a few methods integrate 3-dimensional (3D) structure information. Here, we review one PSSM-based method, i.e. SMM-align (46, 47) and one structure-based method, i.e. MHCII3D (48).

SMM-align (46, 47) predicts quantitative peptide-MHCII binding affinity values. It proceeds by generating a PSSM for a given MHCII allele and optimizing a weight matrix which, when multiplied with the PSSM, reproduces the experimental IC50 values of the binding peptides. Two approaches were used to encode the residues of the peptide sequences: 20 vectors using ‘one hot encoding’, and the substitution score from the Blosum50 matrix. The weight matrix optimization was performed using a Metropolis Monte Carlo procedure (49–51) with the root mean square deviation (RMSD) between predicted and experimental IC50 values of the peptides as cost function. After the training stage, the score of a given 9-residue peptide is obtained by multiplying the weight matrix with the encoded peptide; if for peptides longer than 9 residues, all 9-residue fragments are considered and the highest affinity value is selected.

MHCII3D (48) is the only structure-based predictor that we evaluated. It uses homology modeling to generate scaffold complexes for each distinct MHCII allotype sequence and ensembles of alternative peptide structures that replace the peptide placeholder in the models. The obtained structures of MHCII-peptide complexes, in a simplified backbone representation, were scored using a series of statistical potentials. MHCII3D achieves an AUC of 0.81 which is comparable to sequence-based approaches on a common testing dataset.

#### 2.4.2 Machine-learning based methods

The most successful HLA-peptide interaction prediction methods are based on supervised machine learning (ML) approaches by integrating features extracted from both peptide and MHCII protein sequences. Machine learning algorithms commonly used in these methods are support vector machine (SVM), decision trees (DT) and neural network (NN). Among them, NN is the most widely used and achieves superior performance compared to other algorithms in many HLA-peptide binding prediction studies. In general, the workflow of machine-learning based approaches includes four steps: (1) Construction of training and testing data sets, in which the binding between peptides and MHCII has been verified by experiments; (2) Extraction and selection of features from antigenic peptides, full antigens, MHCII protein sequences and structures; (3) Selection of a suitable ML algorithm and training of the ML prediction model; (4) Optimization of the ML model and evaluation of its performance on an independent dataset. In the following, we list widely used ML-based methods for MHCII-peptide interaction prediction.

In 2019, there were two back-to-back papers published for MHC II-peptide interaction prediction which both combined binding peptide data derived from mass spectrometry (MS) and binding affinity measurement (35, 52). Indeed, the recent developments in MHC II peptidomics allow the identification of MHC II-peptide interactions at a large scale.

MixMHC2pred (35) is one of these two methods that disentangles MS data by a motif deconvolution algorithm (MoDec) for MHCII-peptides prediction (7, 35). MoDec, the core algorithm of MixMHC2pred, is a fully probabilistic framework that does not require the peptide alignment as input and is able to learn multiple motifs at arbitrary positions in the peptide sequences and predict their respective weights as well as binding core position offsets preference. Once MoDec has identified a series of motifs binding with specific alleles using an iterative approach (35), the full predictor then integrates information from allele specific binding motifs, allele-independent peptide N-/C-terminal motifs, peptide length and binding core offset preferences into its final model. It is trained on a MS peptide dataset containing peptides binding with HLA-DR, HLA-DQ and HLA-DP proteins. In the latest version of MixMHC2pred (53), HLAII phosphopeptidome data was used to build a predictor for phosphorylated HLAII ligands.

MARIA (52) is another predictor that uses a multimodal deep neural network to predict the probability of a peptide binding with a specific HLAII protein. The multimodal architecture enables the integration of sequential, categorical, and continuous data into a single deep neural network. Different types of data used for training includes peptide-HLA class II binding pairs derived from liquid chromatography-mass spectrometry (LC-MS) experiments, peptide gene expression data, and *in-vitro* binding affinity. The multimodal deep neural network consists of two major subcomponents: an LSTM recurrent neural network to handle the input peptide sequence of variable length, and a dense neural network to combine the normalized peptide gene expression values (i.e Transcripts Per Million, TPM), a peptide cleavage score and a peptide-HLA class II binding affinity predicted from two pre-trained neural networks. The outer layers of the two major subcomponents are connected through a merge layer that finally computes the probability of the peptide binding to the specified HLA class II allele. MARIA was validated on multiple independent datasets, reaching an area under the curve of 0.89-0.92.

The NetMHCII and NetMHCIIpan predictors (23, 41, 47, 54–61) is a family of predictors that have been developed and upgraded over the past two decades. NetMHCII-1.0 (47), the first version released, is a weight matrix based method that predicts the binding affinity between a peptide sequence and a specific HLA class II allele. It is based on SMM-align which is able to determine the 9-mer core binding motif of a given peptide without the need of sequence alignment and instead simply extracts the 9-mer with the highest predicted binding affinity both during training and inference. SMM-align was trained on ∼ 5.000 peptide-HLA (pHLA) quantitative binding affinity values across 17 different HLA alleles. The allele-specific weight matrices were optimized to reproduce the corresponding experimental IC50 values by a Monte Caro search.

One of the limitations of NetMHCII-1.0 was that users could only select among the HLA class II alleles that were available in the training dataset. To overcome this limitation, it was replaced by a neural network-based pan HLA-DR predictor named NetMHCIIpan-1.0 (55). To enable the prediction of HLA-DR alleles that are not part of the training dataset, the authors developed the “HLA Pseudo-Sequence”, which is a sequence of 21 residues that represents the binding groove of MHCII receptors and was determined after a careful analysis of 15 peptide-MHCII 3D protein structures. The peptide and HLA pseudo-sequence residues are encoded using one-hot encoding and BLOSUM50 substitution matrix encoding, respectively, meaning each residue requires 20 input neurons. In total, the neural network has 658 input neurons. The model was trained on ∼ 14.600 pHLA binding affinity data points across 14 human HLA-DR alleles.

NetMHCII-2.0 (56), which was published soon after as an update of NetMHCII-1.0, is largely inspired by the neural network approach used in NetMHCIIpan-1.0 described above. As a result, SMM-align was replaced by NN-align, which essentially expands on the SMM-align method but replaces the weight matrices with a neural network. As in SMM-align, NN-align determines the 9–mer core binding motif of a peptide by identifying the one with the best binding affinity both during training of the neural network and inference.

NetMHCIIpan-2.0 (57) was released shortly after. It essentially updated the previous version by replacing SMM-align with NN-align and also updated the training dataset to ∼ 34.000 pHLA binding affinity data points covering 24 HLA-DR alleles. The neural network architecture and input features remain exactly the same as in NetMHCIIpan-1.0. On a number of different benchmarks, the method reached an average AUC across different HLA-DR alleles of between 0.78 and 0.85.

Three years later, NetMHCIIpan-3.0 was released (58), which is the first truly pan specific method as, in addition to HLA-DR alleles, it is capable of predicting pHLA binding affinity for any HLA-DP and HLA-DQ alleles. This was achieved by generalizing the concept of the HLA pseudo-sequence to all three HLA allele isotypes, which resulted in a new definition of the HLA pseudo-sequence that includes 34 binding residues instead of 21. The training dataset was also updated to ∼ 52.000 pHLA binding affinity data points.

In 2018, NetMHCII-2.3 (41) and NetMHCIIpan-3.2 (41) were released following the update of the training datasets of both methods with a new training dataset containing ∼ 134.000 pHLA binding affinity data points. This update slightly increased the performance of the methods, with an average AUC-ROC across all alleles of 0.76 - 0.86 for NetMHCII-2.3 and 0.78 - 0.86 for NetMHCIIpan-3.2.

A major update of NN-align was published in 2019 (60) which enabled the method to be able to deal both with pHLA binding affinity data as well as LC-MS data. LC-MS data is inherently poly-specific, which means it returns a set of multiple HLA alleles and peptides that bind to them, but the exact pairing of each pHLA is unknown. To deconvolute this data and obtain pHLA pairs, they developed NN-align MA (Multi Allele). Briefly, NN-align was adapted by first training the neural network for a number of iterations on Single Allele (SA) data (i.e binding affinity pHLA data and some SA LC-MS experimental data) and the resulting model was used to annotate and thus deconvolute the MA LC-MS data. All the data is then combined and used to train the neural network. The latest pan allelic method, NetMHCIIpan-4.1 (61) used the newly developed NN align MA algorithm and a training dataset of *>* 500.000 binding affinity and LC-MS pHLA data, reaching a median ROC-AUC of 0.98 in cross-validation.

BERTMHC (62) uses a bidirectional encoder representation from transformers (BERT, Devlin et al., 2019) (63) to predict the binding affinity (real values) and binding probability (binary) of a given peptide and HLA class II allele. The method encodes each amino acid as a 768-dimensional vector using the protein language model TAPE (64) which was pre-trained using over 31 million protein sequences from the Pfam database. The input sequence was learned by 12 self-attention layers and the outputs of each layer were concatenated to give final vectors. Two BERT models were trained, one for binding affinity and one binary binding prediction, respectively. For the binary predictor, deconvolution was performed using Multiple Instance Learning (MIL) on the LS-MS data in which one peptide could bind with several HLA proteins (65). The authors performed multiple evaluation tests reaching AUC values *>* 0.95 for binding prediction and of 0.72 for binding affinity prediction (Pearson correlation = 0.39).

MHCnuggets (66) is an allele-specific peptide-HLA binding predictor that uses an LSTM recurrent neural network to predict the binding probability for peptide-HLAI interactions and binding affinity for peptide-HLAII proteins. In order to handle the sequence length variability, padding is added to the peptide sequences so that they all reach a size of 15 or 30, respectively for HLA class I and II. In total, 136 HLA class II predictors were trained, one for each allele. To improve the performance of the predictors with limited training data available, a transfer learning approach was used. Briefly, the HLAII alleles with the most abundant data were trained first, and the resulting weights were used to initialize the other allele-specific predictors. The method reaches an average AUC of 0.85 in cross-validation.

#### 2.4.3 Consensus methods

Consensus-based MHC-peptide prediction methods integrate the outputs of several prediction tools through weighting schemes with the aim of obtaining better predictions than any individual method. The most commonly used consensus method is IEDB Consensus (37, 67), which is the tool recommended by the IEDB platform and combines NN-align, SMM-align and CombLib (67, 68).

### 2.5 Webserver/software functionality

We checked the availability of all methods listed in **Table 1**. Eleven methods could be run by webserver and/or a standalone package which were selected for further evaluation of prediction performance. These predictors are: MHCnuggets, MARIA, MixMHC2pred, BERTMHC, NetMHCIIpan-4.1, NetMHCIIpan-4.0, NetMHCII-2.3, NetMHCIIpan-3.2, NetMHCII, MHCII3D and IEDB consensus. Most of the methods are based on NN (we already introduced earlier) except NetMHCII and MHCII3D, which employ scoring function approaches, and IEDB consensus methods. One should note that not all the methods can predict peptides for any lengths and MHCII alleles. Indeed, some methods are limited to identify peptides with specific lengths and certain MHCII alleles. Out of all eleven approaches, only NetMHCIIpan-3.2, NetMHCIIpan4.0, NetMHCIIpan-4.1 and MHCnuggets could predict the probability score for all the peptides in our benchmark dataset. Software availability details are shown in **Table 1**.

## 3 Results and discussion

### 3.1 Conservation analysis of sequence motifs of the MHCII binding peptides

To analyze to what extent the peptides that bind to a specific HLAII allele have similar sequences, we aligned these peptides without considering their length as described in Methods section 2.3. We identified from these alignments the 9-mer regions of the peptides as those that have the least percentage of gaps.

**Figure 3:**
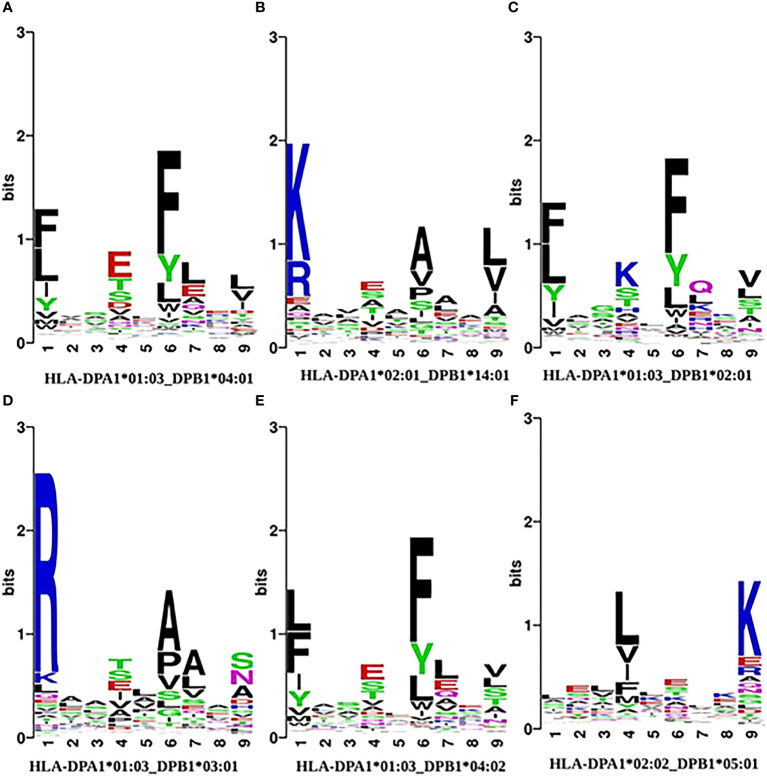
Analysis of the amino acid positional preferences for allotype-specific peptide ligands in specific HLAII allotypes: **(A)** HLA-DPA1*01:03 - DPB1*04:01,**(B)** HLA-DPA1*02:01-DPB1*14:01, **(C)** HLA-DPA1*01:03-DPB1*02:01, **(D)** HLA-DPA1*01:03-DPB1*03:01, **(E)** HLA-DPA1*01:03-DPB1*04:02, **(F)** HLA-DPA1*02:02-DPB1*05:01. The motifs are aligned with respect to the center of the 9-mer binding core. The overall height of letters indicates the sequence conservation at that position, while the height of each amino acid represents the relative frequency of that residue.

We then analyzed the position-dependent amino acid preferences of peptides and generated allele-specific plots using the seqlogo program of WebLogo (69). In **Figure 3**, we show examples of the conservation logo of peptides binding with six different HLAII alleles. The plots for other alleles are available in **Supplementary Data S5**. In general, aligned peptides show a conserved pattern characterized by anchors at positions P1, P4, P6, and P9, and sometimes also P7, except HLA-DPA1*02:02 DPB1*05:01 which shows conservation only in P4 and P9.

HLA-DPA1*01:03_DPB1*04:01 has the largest number of binding peptides in our benchmark dataset (**Figure 3A**). We observed that the binding peptides show a preference for Phe (F) or other aromatic or hydrophobic amino acids such as Leu (L) at positions P1 and P6, which is consistent with earlier observations (70). Moreover, there is a preference for Glu (E) at position P4 and Leu (L) at P9. These four positions constitute the anchor residues of this HLA protein. Similar patterns were found for HLA-DPA1*01:03_DPB1*02:01 (**Figure 3C**) and HLA-DPA1*01:03_DPB1*04:02 (**Figure 3E**) except that in the former the preference for Glu at P4 is replaced by a preference in Lys (K) at position P4. Position P7 also slightly differs.

Peptides binding to HLA-DPA1*02:01_DPB1*14:01 show a preference for the positively charged residues Lys (K) and Arg (R) at position P1, Glu (E) and Ser (S) at P4, Ala (A) and Val (V) at P6 and Leu (L) and Val (V) at the position P9. In contrast, the peptides that bind to HLA-DPA1*02:02_DPB1*05:01 have essentially two conserved positions only: Leu/Val (L/V) at position P4 and Lys (K) at position P9.

For a recent comprehensive investigation of the MHCII binding motif preferences for about 600k peptides identified by MS and bound to more than eighty MHCII alleles in human, mouse, cattle and chicken, see (71).

### 3.2 Performance evaluation of peptide-MHCII binding predictors

**Figure 4:**
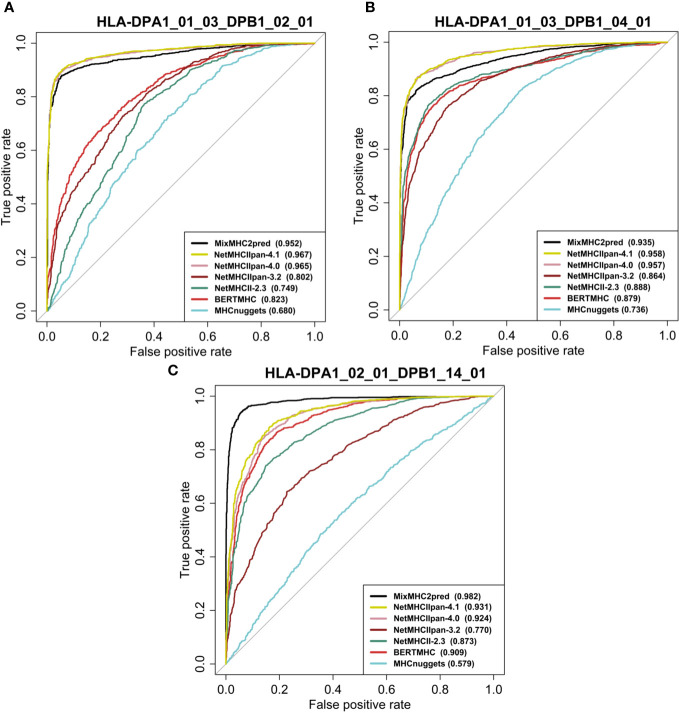
Performance of eleven MHC class II prediction methods assessed by their ROC curves and AUC values. The curves were generated by plotting the true positive rate (y-axis) against the false positive rate (x-axis). ROC curves for peptides binding to HLAII molecules specific for **(A)** HLA-DPA1*01:03-DPB1*02:01 (15mer), **(B)** HLA-DPA1*01:03-DPB1*04:01 (15mer) and **(C)** HLA-DPA1*02:01-DPB1*14:01 (15mer). AUC values for each prediction method are provided between parentheses in the subfigure legends. For prediction results on other HLA classes and peptide lengths see **Supplementary Figure S1** and **Data S4**.

**Figure 5:**
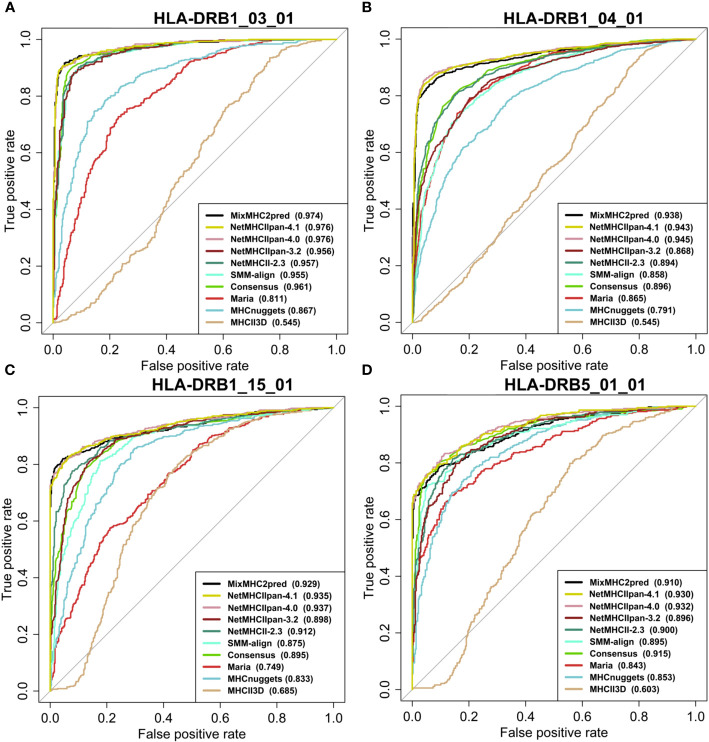
Performance of MHC class II prediction methods for HLA-DR genes assessed by their ROC curves and AUC values. ROC curves for peptides binding to HLAII molecules specific for **(A)** HLA-DRB1*03:01 (15mer), **(B)** HLA-DRB1*04:01 (15mer), **(C)** HLA-DRB1*15:01(15mer), **(D)** HLA-DRB5*01:01 (15mer). AUROC values for each prediction method are provided between parentheses in the subfigure legends. For prediction results on other HLA classes and peptide lengths see **Supplementary Figure S1** and **Data S4**.

We assessed the performance of the eleven available predictors on our independent test dataset. For all peptides with a certain length that binds to a specific MHCII allele, we computed the predictions with the different methods and plotted the ROC curves of each predictor. **Figures 4**, **5** show examples of ROC curves for the predictions of MHC-peptide binding for the tested methods. Here we selected the subsets of MHCII-peptide interactions containing the largest numbers of peptides. All prediction outputs of each method can be seen in **Supplementary Data S2** and all AUCs calculated are in **Supplementary Data S3**.

As can be seen in the figures, most of the methods tested perform well in predicting MHCII-peptide binding. In general, ML-based methods achieved better performances across almost all 20 allotypes than scoring-based and consensus approaches. Among them, MixMHC2pred and NetMHCIIpan-4.1 performed the best, with the highest ROC-AUC scores. Moreover, NetMHCIIpan-4.1 is able to predict peptides with a wider length range (10-19 amino acids) and covers more allotypes (**Supplementary Data S3**).

Interestingly, IEDB consensus methods perform rather well on several MHCII-peptide subsets, especially on HLA-DRB isoforms. The average AUC reaches 0.84 ± 0.12. The two best methods, MixMHC2pred and NetMHCIIpan-4.1, are able to predict most peptide-MHCII interactions with good accuracy and we observe some complementarity between them. For example, NetMHCIIpan-4.1 is able to predict 11-residue long peptides (AUC equal to 0.876 ± 0.104), while MixMHC2pred outputs no results. In contrast, MixMHC2pred achieved better performances on 12-residue peptides binding to HLA-DPA1_02_01_DPB1_01_01 than NetMHCIIpan-4.1, with an AUC of 0.985 and 0.820, respectively. One can thus expect that a consensus approach combining these two predictors will achieve better accuracy.

Based on the performance on our independent datasets, we see a step-by-step improvement of the NetMHCII predictor family from the older to the newer versions. Indeed, the average AUC of NetMHCIIpan-3.2 reaches 0.793± 0.121 and that of NetMHCII2.3, 0.844 ± 0.124. NetMHCIIpan-4.0 increases the score up to 0.932 ± 0.06 and NetMHCIIpan-4.1, to 0.936 ± 0.06. NetMHCIIpan-3.2 is able to cover peptides with a wider length range and more MHCII alleles than NetMHCII-2.3, but the latter obtained a better performance than NetMHCIIpan-3.2. NetMHCIIpan-4.1 and NetMHCIIpan-4.0 achieve significantly superior performance than most methods on all HLA alleles. This is probably enabled by the use of a larger training set composed of the MHC ligands eluted by MS and binding affinity datasets from IEDB. Another key point is the implementation of a more complex machine learning framework to extract features from peptide sequences and MHC proteins and also the deconvolution of the multi allele LC-MS data in NetMHCIIpan-4.1. Similarly to NetMHCIIpan-4.1, the other best approach, MixMHC2pred, also took the MS ligand and binding affinity datasets as training sets and used the MoDec framework to perform motif deconvolution. Both strategies significantly boost the performance of MHCII-peptide interaction prediction.

The PSSM-based method SMM-align, developed over fifteen years ago, performs surprisingly well for MHC class II binding prediction (AUC 0.828 ± 0.114). However, it only works for a small number of HLA-DR and DP genes which greatly limit its clinical applications due to the shortage of algorithms coping with variable length of peptides.

Furthermore, we calculated the maximum BACC scores for each method for the whole dataset and each subset of given peptide length and HLA allotype by optimizing the cut-offs of the binary prediction (**Supplementary Table S1**). For the full dataset, MixMHC2pred obtained the best maximum BACC score (0.906), while NetMHCIIpan4.0 and NetMHCIIpan4.1 also achieved nice results (0.858 and 0.850, respectively). We compared these best BACC scores with the scores calculated using the default cut-offs provided by the webserver or local programs. All the optimal BACC scores are slightly higher than the default ones (average difference of 0.033 ± 0.014). However, for certain HLA allotypes or peptide lengths, the optimized BACC is substantially higher than the default one, which suggests that the default cut-offs are in some cases sub-optimal. Our BACC score and cut-off analyses will facilitate the choice of the adequate predictor for given peptide length binding with given HLAII protein. The best BACC scores, cut-offs and types of prediction scores used for each method are also listed in **Supplementary Data S4** which also contains the best F1 score as an additional metric.

In conclusion, our results show that the most recent predictors are in general better than methods developed a long time ago, which may be due to the larger amount of training data and more advanced machine learning architectures. Also, we showed that the default thresholds suggested by the methods work well for most alleles, but are suboptimal for a subset of alleles, which suggests the need for method developers to provide allele-specific thresholds instead of a single threshold for all alleles.

### 3.3 DR, DP and DQ alleles

The IEDB database contains HLA-DP, HLA-DQ, and HLA-DR data but it is dominated by the latter allele. HLA-DRs have been widely investigated given their prominent role in the immune system. Our benchmark dataset is characterized by a higher proportion of HLA-DP due to the redundancy removing process during data preprocessing (see Methods section). Thanks to the recent increase in the amount of available data, DP alleles have also been found to be crucial for many diseases, such as primary sclerosing cholangitis (72), ulcerative colitis (73), acute lymphoblastic leukemia (74), hepatitis B virus infection (75) and cervical cancer (76). Although HLA-DQ data are less abundant, this allele is nonetheless important in some autoimmune disorders such as celiac disease (77). It is thus important to have benchmark datasets such as ours to comprehensively assess MHCII-peptide binding predictors on all three HLA alleles separately.

Furthermore, whereas some predictors are only able to predict peptide binding to DR allele, the majority of the methods, among which the pan-allele predictors NetMHCIIpan-4.1 and MixMHC2pred, have been developed to cope with DP, DQ, and DR alleles, as shown in **Table 1**.

For these reasons, we evaluated the method performances separately on *DRset_bench_*, *DPset_bench_* and *DQset_bench_* and show the results in **Table 2** and **Supplementary Table S2**. It is noteworthy that predictors perform a little bit better on DR alleles than on DP and DQ. This is probably due to the fact that DR data is more abundant in their training datasets than other types of alleles. Top methods are in any case able to achieve very good performances on the three alleles, with MixMHC2pred performing best on DP and DQ alleles, whereas NetMHCIIpan-4.0, NetMHCIIpan-4.1 and MixMHC2pred all obtained AUC scores of about 0.96 on DR data.

**Table 2:** Performance of the prediction of peptides binding with either DP, DR or DQ alleles.

DR		DP		DQ	
Predictor	AUC	Predictor	AUC	Predictor	AUC
NetMHCIIpan-4.0	0.963	MixMHC2pred	0.961	MixMHC2pred	0.941
NetMHCIIpan-4.1	0.962	NetMHCIIpan-4.1	0.918	NetMHCIIpan-4.1	0.892
MixMHC2pred	0.956	NetMHCIIpan-4.0	0.909	NetMHCIIpan-4.0	0.890
IEDB consensus	0.893	BERTMHC	0.820	NetMHCII-2.3	0.881
NetMHCII	0.885	NetMHCII-2.3	0.804	BERTMHC	0.878
NetMHCII-2.3	0.880	NetMHCIIpan-3.2	0.740	MARIA	0.873
NetMHCIIpan-3.2	0.875	NetMHCII	0.715	NetMHCII	0.836
MARIA	0.839	IEDB consensus	0.682	NetMHCIIpan-3.2	0.793
MHCnuggets	0.747	MHCnuggets	0.648	MHCnuggets	0.703
MHCII3D	0.519	MARIA	N/A*	IEDB consensus	0.657
BERTMHC	N/A*	MHCII3D	N/A*	MHCII3D	N/A*

*BERTMHC needs to specify both the α and β chains of DR alleles; MHCII3D works for DR but not DP and DQ alleles; MARIA can predict peptides binding with DR and DQ, but not DP.

### 3.4 Current methods and prospective strategies

In the past decade, computational approaches predicting peptides binding to MHCII molecules have witnessed a rapid development and are becoming increasingly attractive and useful for biomedical research. In particular, newer methods such as MARIA, MixMHC2pred and NetMHCIIpan-4.1 have been developed since 2019 and now integrate data from MS, which has significantly boosted their predictive accuracy for peptide binding prediction. However, predicting MHCII-peptide binding is still a challenging problem. Firstly, the datasets used to train and test these approaches only cover a very small fraction of all MHCII proteins coupled with variations of MHCII allele. Although our benchmark dataset includes all five types of HLA-DRA proteins, there are over 2500 HLA-DRB and 1400 HLA-DQB proteins according to IMGT’s database (78). In IEDB, which is the largest binding peptide database, most studies still focus on a limited number of MHCII proteins. This might bias these predictors towards accurately predicting peptides binding with certain types of MHCII proteins only. Secondly, every patient is highly personalized, in particular in the context of cancer patients. Adding personalized characteristics, disease details and quantitative information of MHC-peptide binding could not only improve the predictors but also help in the development of personalized cancer vaccines and immunotherapy. With more data available as a result of high-throughput experimental methods and the availability of detailed patient-specific medical information, one could expect these computational approaches to get closer to solving these problems. Thirdly, most methods are trained on human peptides, but in the near future, new MHCII-peptide binding predictors able to interpret non-human peptides such as microbiome could provide new immunotherapeutic options for infectious diseases.

The majority of current approaches are sequence-based and neglect the structural properties of MHC proteins and peptides. Structural features such as the stability of HLA-peptide complexes, structural details of binding grooves and the structural contacts between MHC protein and peptides have proved to be crucial for protein-peptide recognition and peptide drug development. Moreover, the recent development of protein structure prediction approaches, such as AlphaFold2, has greatly improved the accuracy of protein 3D structure prediction (79). In the near future, machine-learning approaches integrating structural features with sequence properties could further improve the predictive power of these methods and more importantly, deepen the understanding of the complex binding mechanism of MHC-peptide complex.

## 4 Conclusions

In this paper, we have reviewed eleven available tools for predicting peptides binding with MHCII molecules. By constructing an independent and well curated dataset covering all three HLA alleles, we evaluated state-of-the-art MHCII-peptide binding predictors and underlined their strengths and weaknesses. Our study provides a useful guide for researchers interested in using the best predictor for their use case as well as in improving existing methods and developing new ones. Moreover, it will be helpful to academic and industrial researchers working on tumor vaccine development and drug design.

## Author contributions

YY: Conceptualization, Data curation, Formal Analysis, Investigation, Methodology, Project administration, Resources, Validation, Writing – original draft. ZW: Data curation, Formal Analysis, Investigation, Methodology, Validation, Writing – original draft. GC: Formal Analysis, Investigation, Methodology, Writing – original draft, Project administration. XS: Investigation, Methodology, Project administration, Writing – original draft. FP: Investigation, Methodology, Project administration, Writing – original draft, Conceptualization, Data curation, Funding acquisition, Supervision. MR: Conceptualization, Data curation, Funding acquisition, Project administration, Supervision, Writing – original draft. FX: Funding acquisition, Project administration, Supervision, Writing – original draft, Formal Analysis. QH: Formal Analysis, Funding acquisition, Project administration, Supervision, Writing – original draft, Conceptualization, Data curation, Investigation, Methodology, Resources, Software, Validation, Visualization, Writing – review & editing.
