# Supplementary material for: MHCII-peptide presentation: an assessment of the state-of-the-art prediction methods
Source: Front Immunol. 2024 Mar 12;15:1293706. doi: 10.3389/fimmu.2024.1293706 (PMC11027168; doi:10.3389/fimmu.2024.1293706)
Supplement: Supplementary file 5 [file DataSheet_5.docx]

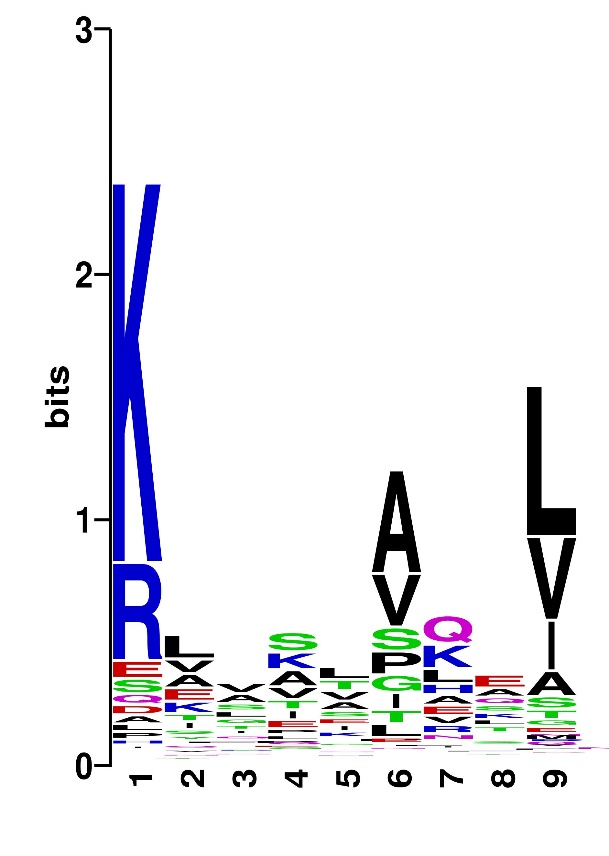

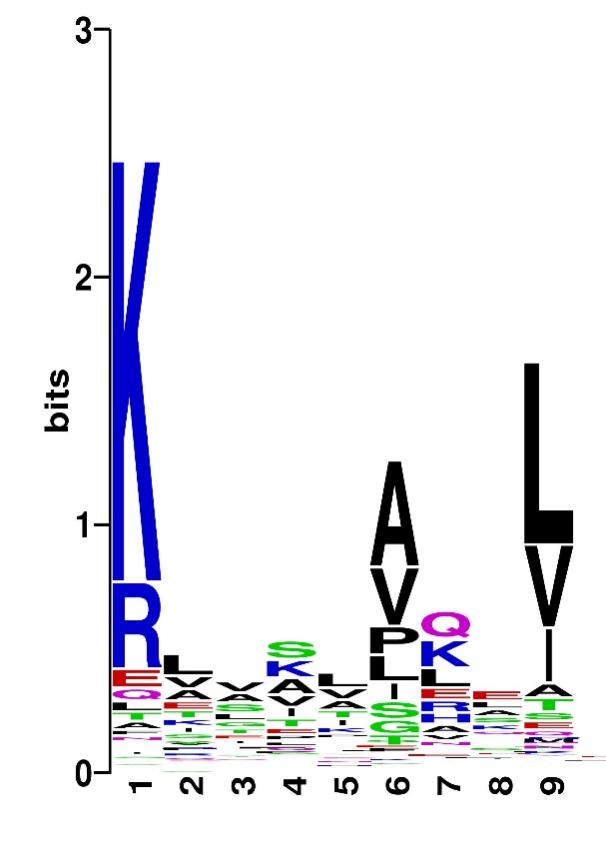

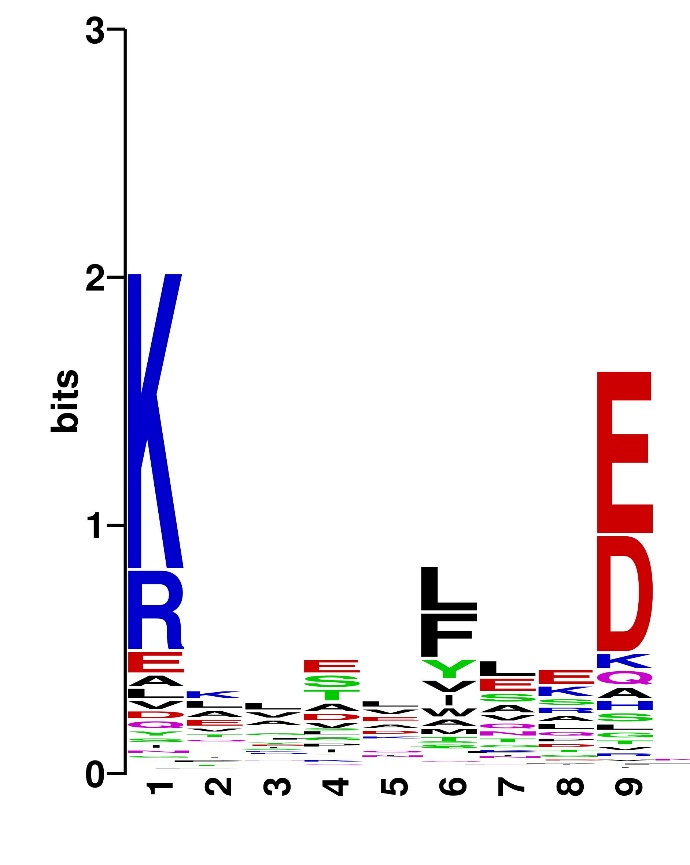


**I**

**H**

**G**

**HLA-DPA1*02:01_DPB1*01:01**

**HLA-DPA1*02:01_DPB1*09:01**

**HLA-DPA1*02:01_DPB1*10:01**


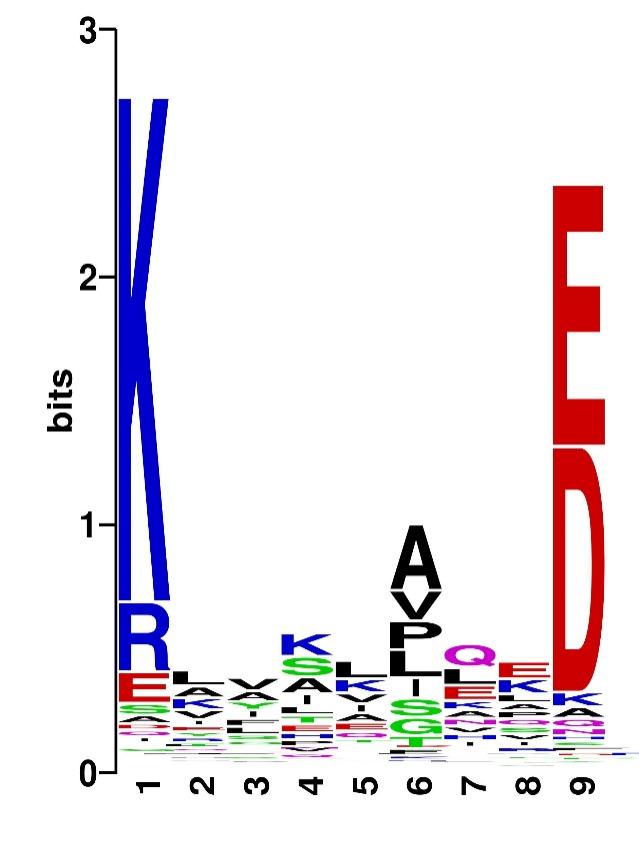

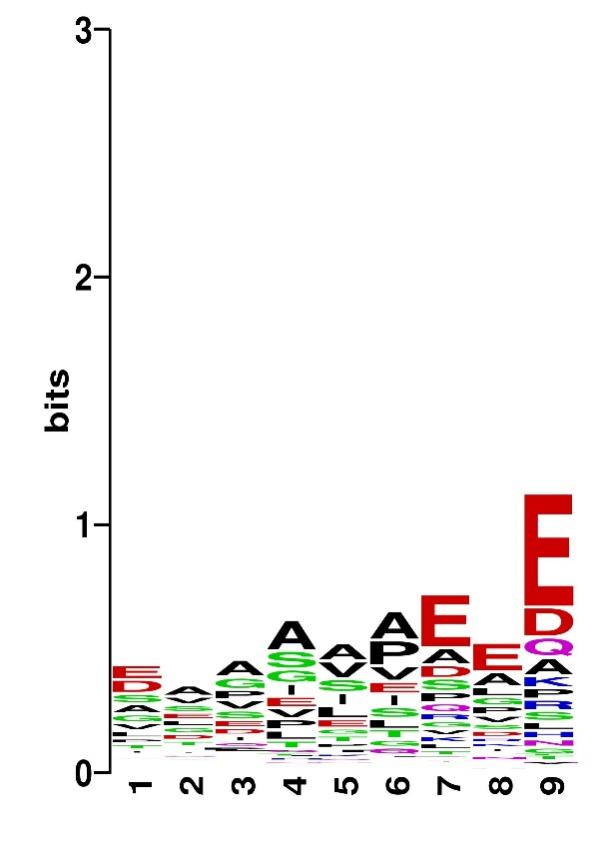

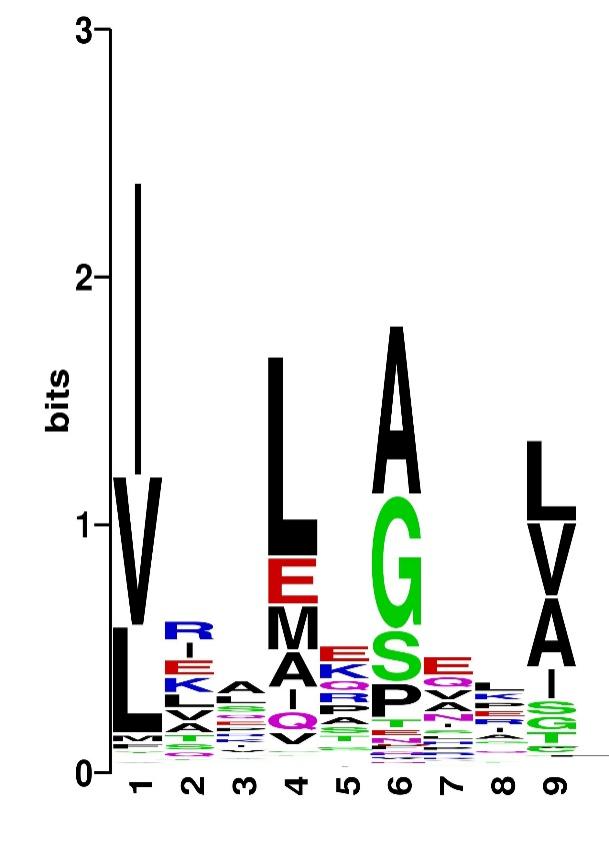


**L**

**K**

**J**

**HLA-DRB1*01:02**

**HLA-DQA1*03:01_DQA1*03:02**

**HLA-DPA1*02:01_DPB1*13:01**


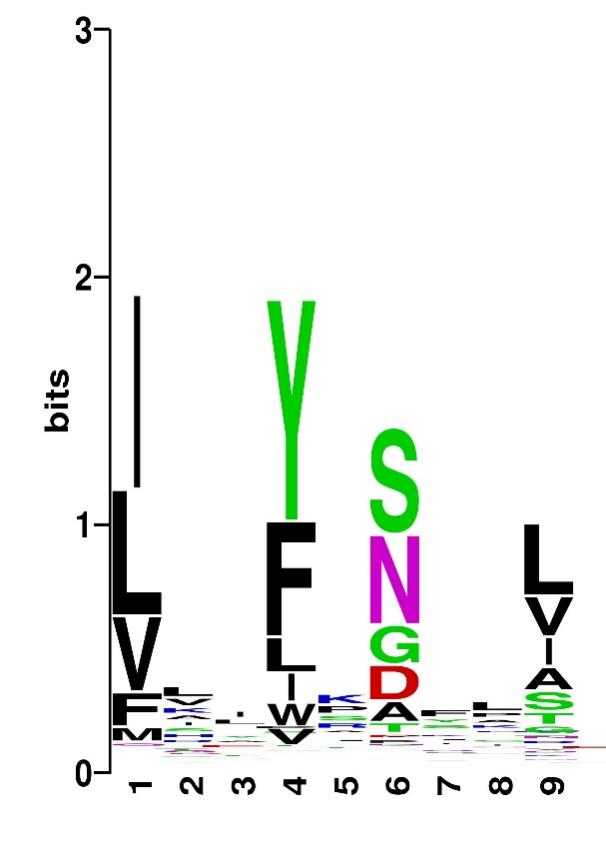

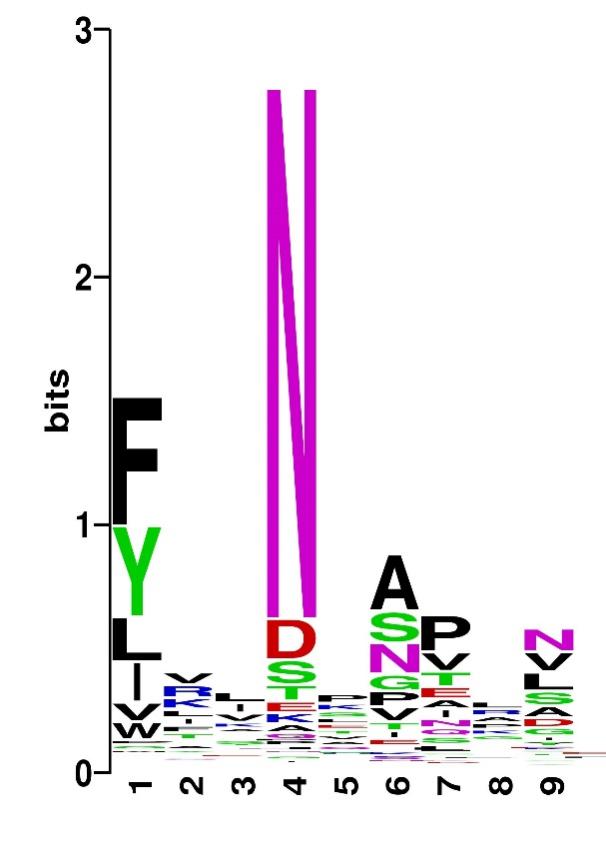

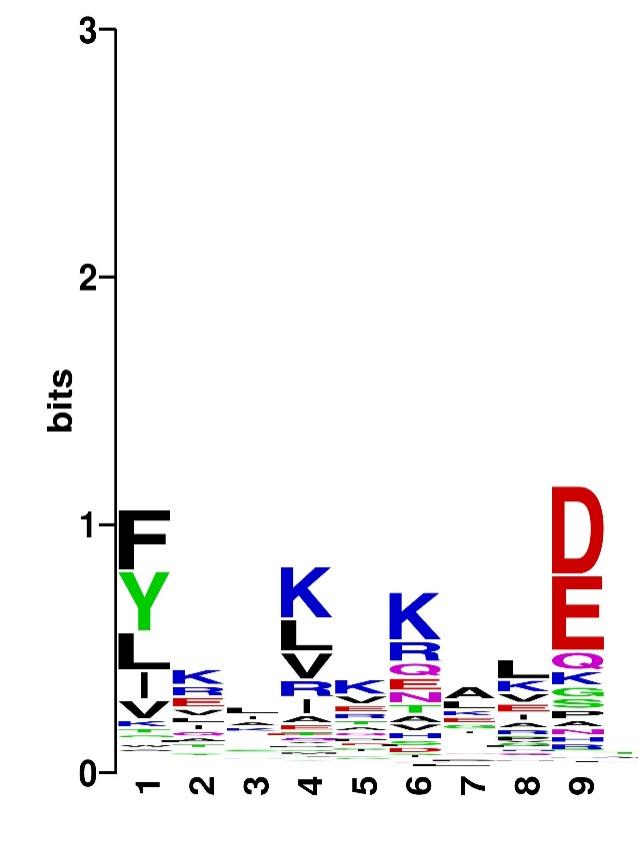

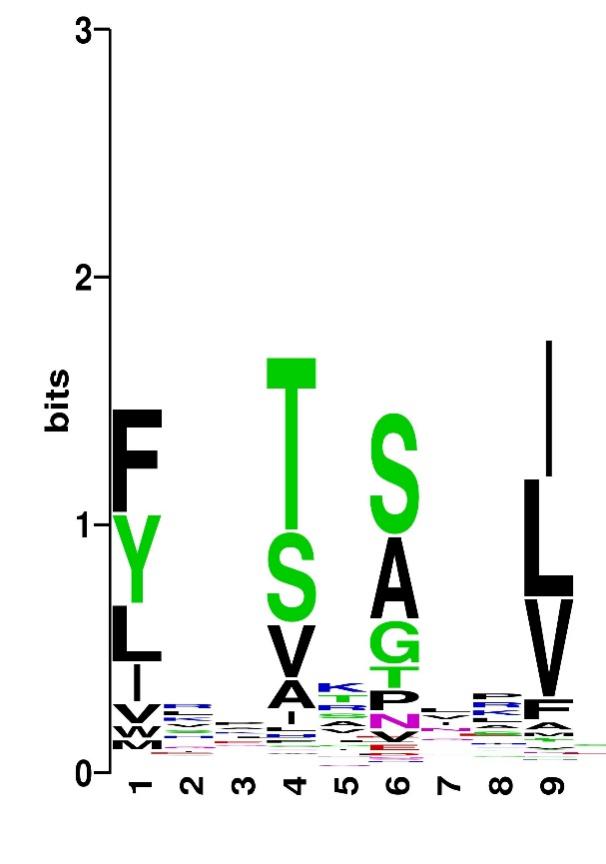

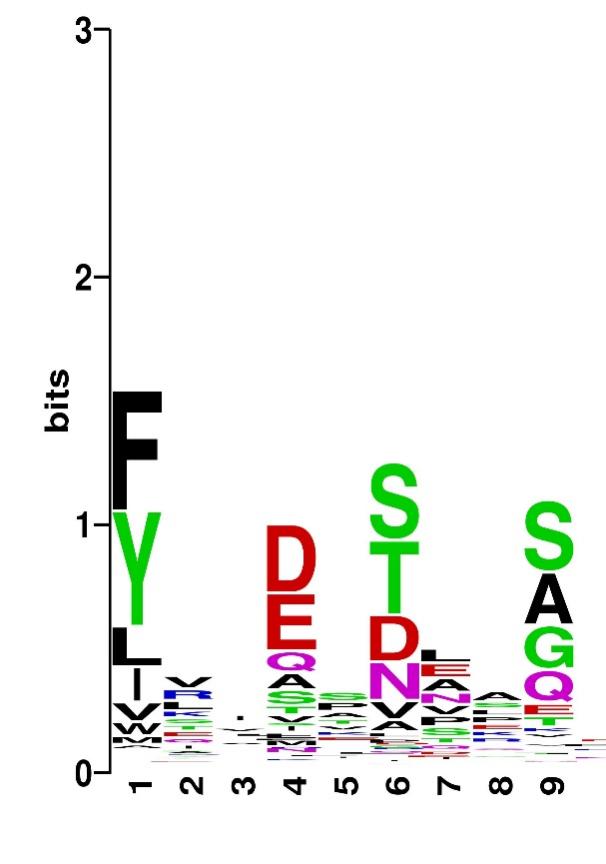

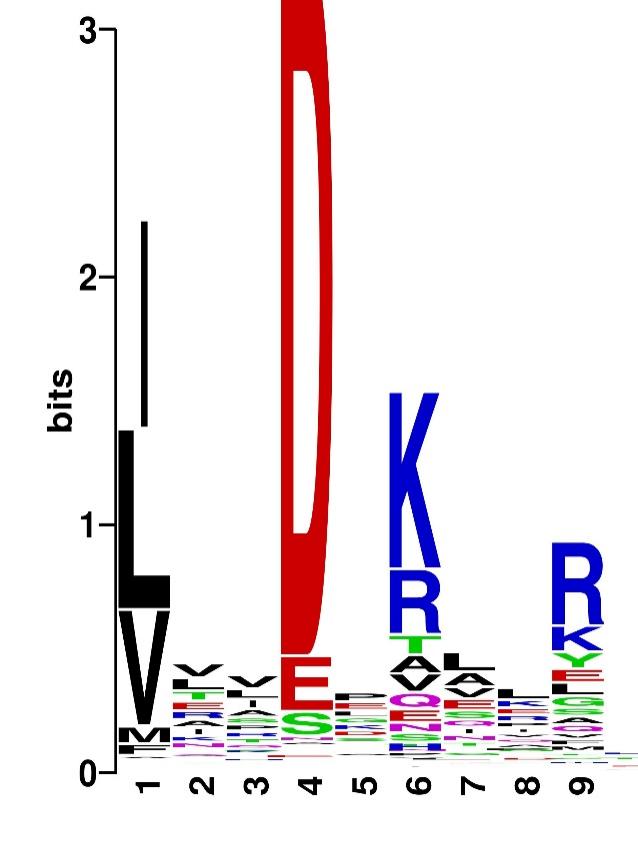

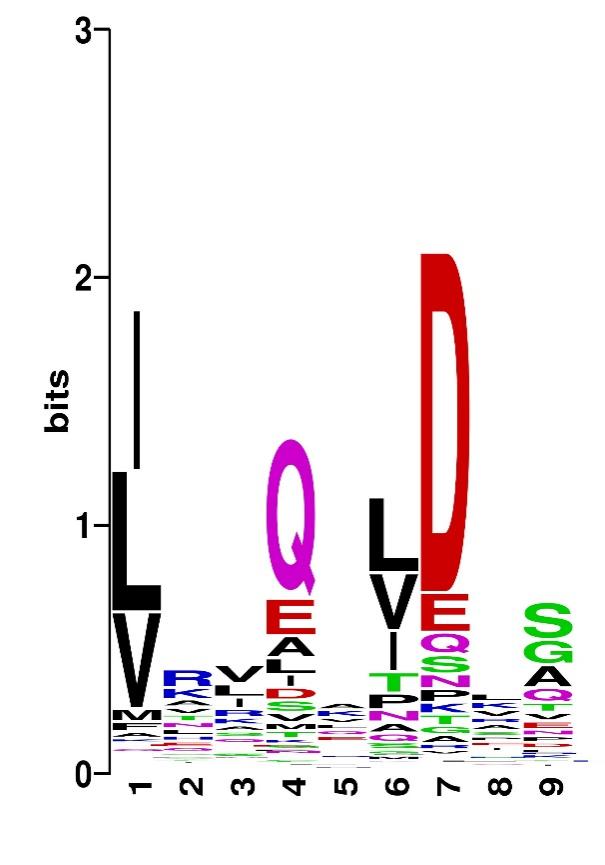

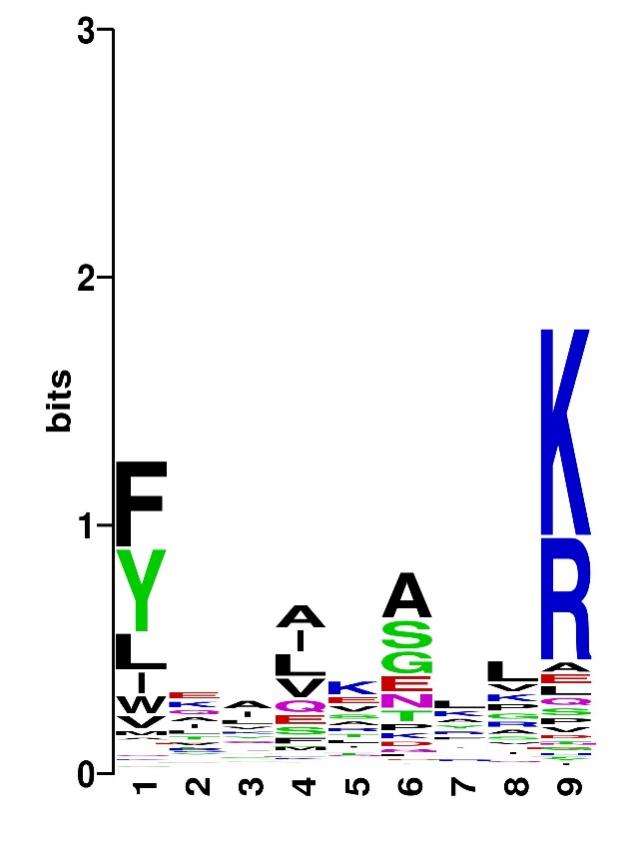


**R**

**Q**

**P**

**O**

**N**

**M**

**HLA-DRB3*02:02**

**:02**

**HLA-DRB1*15:01**

**HLA-DRB1*08:01**

**HLA-DRB1*07:01**

**HLA-DRB1*04:01**

**HLA-DRB1*03:01**

**S**

**G**

**HLA-DRB4*01:03**

**HLA-DRB5*01:01**

**:02**


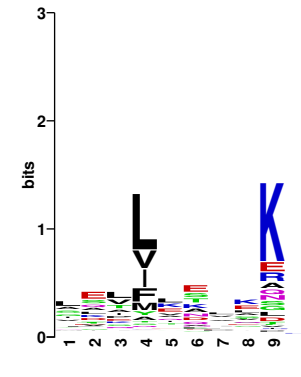

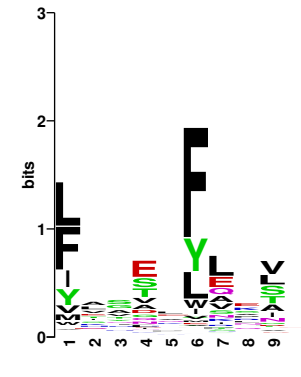

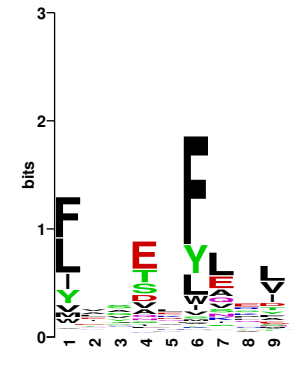

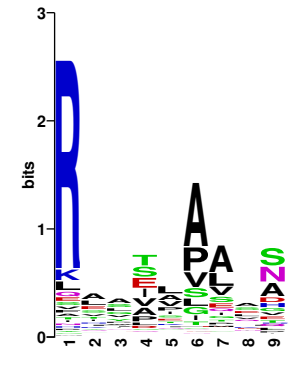

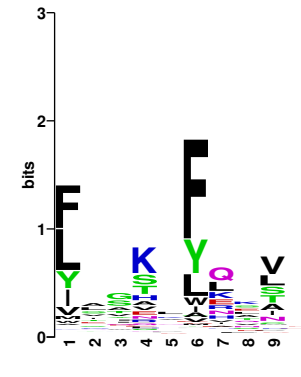

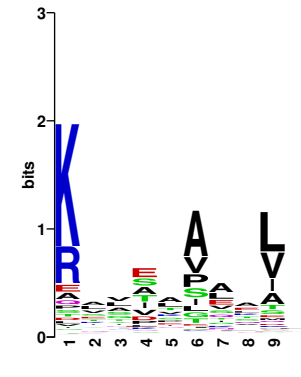


**HLA-DPA1*02:02_DPB1*05:01**

**HLA-DPA1*01:03_DPB1*04:02**

**HLA-DPA1*01:03_DPB1*02:01**

**HLA-DPA1*01:03_DPB1*04:01**

**HLA-DPA1*02:01_DPB1*14:01**

**F**

**E**

**D**

**C**

**B**

**A**

**HLA-DPA1*01:03_DPB1*03:01**
