# Supplementary material for: MHCII-peptide presentation: an assessment of the state-of-the-art prediction methods
Source: Front Immunol. 2024 Mar 12;15:1293706. doi: 10.3389/fimmu.2024.1293706 (PMC11027168; doi:10.3389/fimmu.2024.1293706)
Supplement: Supplementary file 6 [file DataSheet_6.pdf]

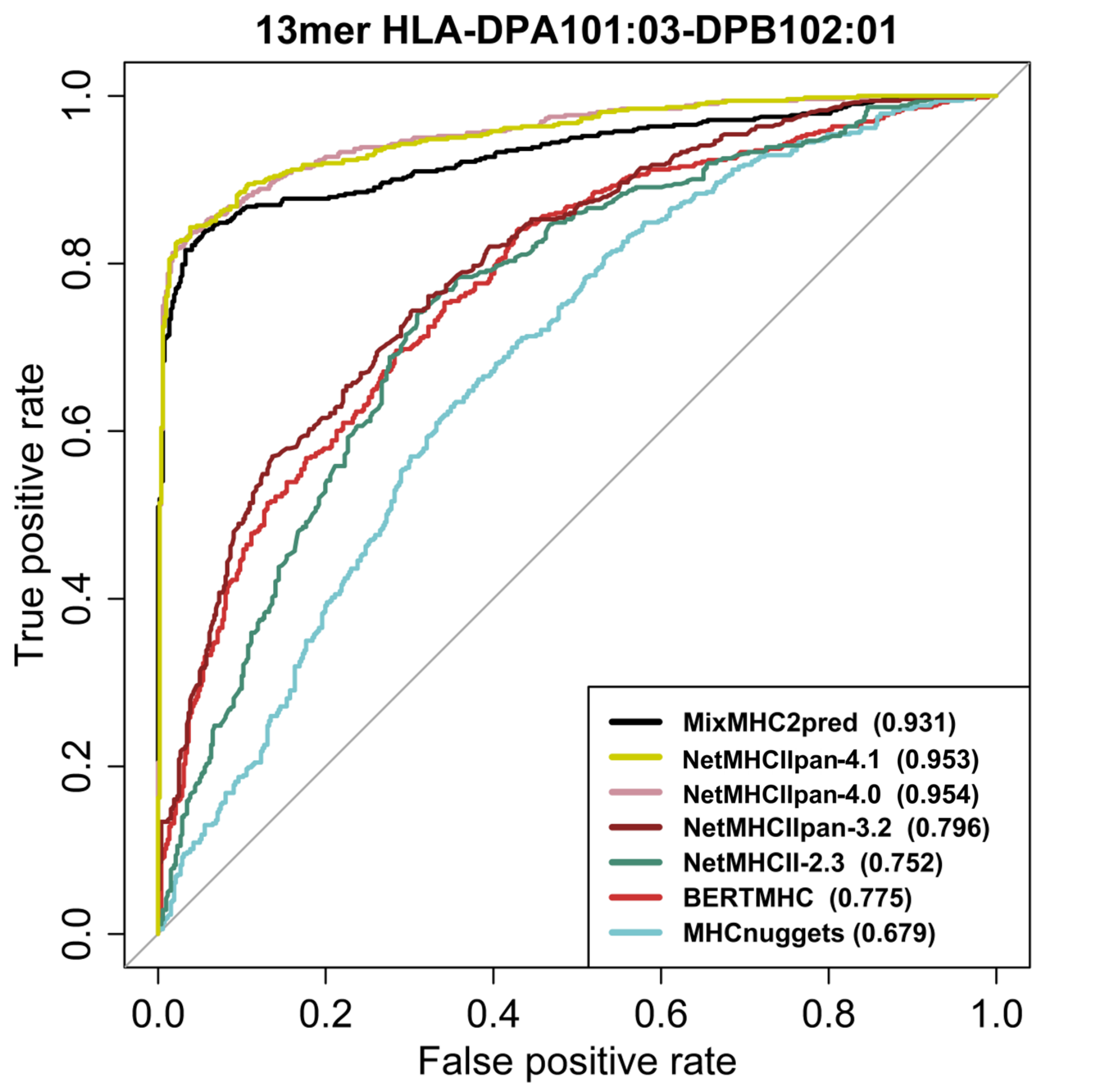
13mer HLA-DPA101:03-DPB102:01
True positive rate
False positive rate
0.0
0.2
0.4
0.6
0.8
1.0
MixMHC2pred (0.931)
NetMHCIIpan-4.1 (0.953)
NetMHCIIpan-4.0 (0.954)
NetMHCIIpan-3.2 (0.796)
NetMHCII-2.3 (0.752)
BERTMHC (0.775)
MHCnuggets (0.679)

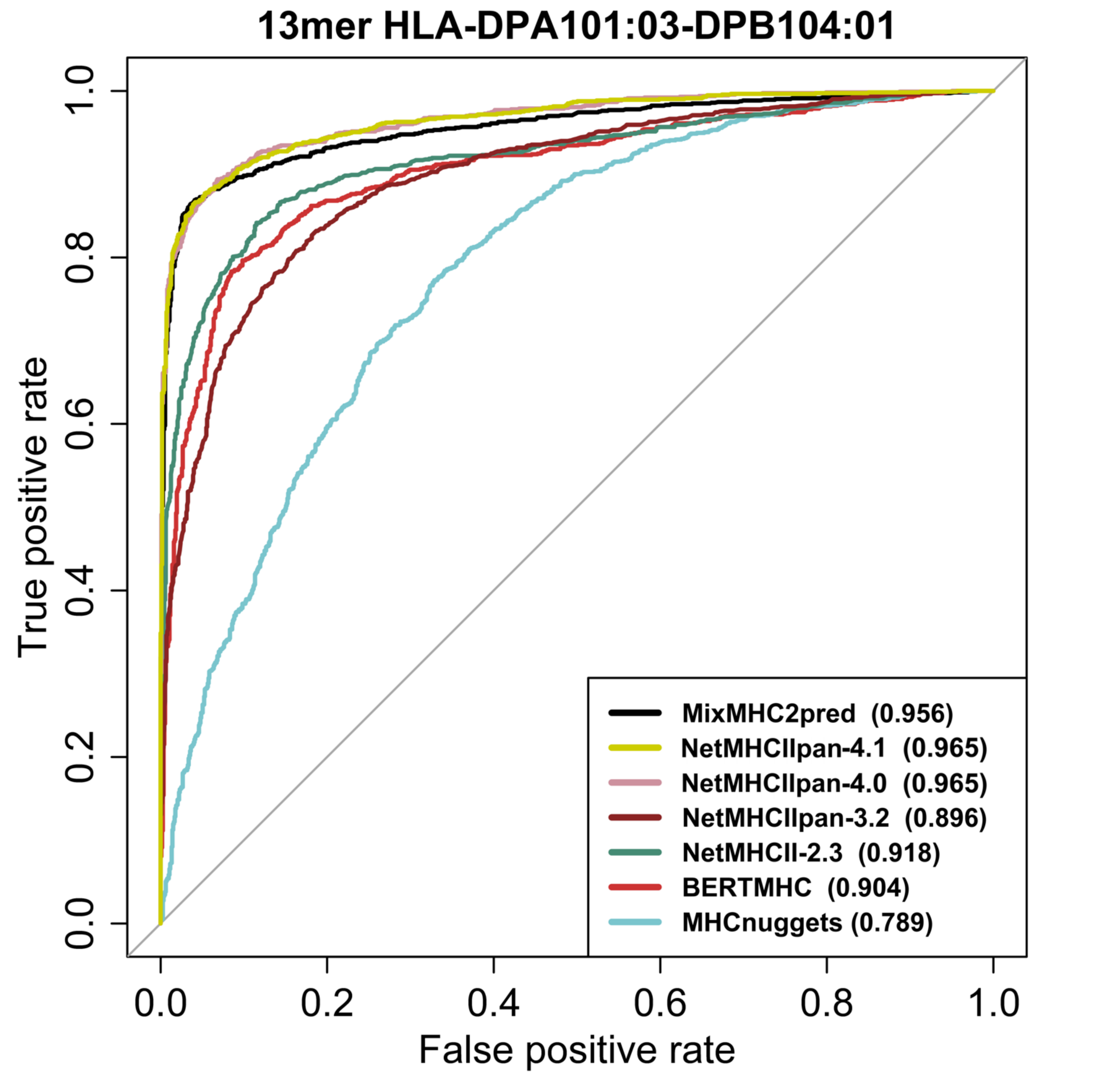
13mer HLA-DPA101:03-DPB104:01
True positive rate
False positive rate
0.0
0.2
0.4
0.6
0.8
1.0
MixMHC2pred (0.956)
NetMHCIIpan-4.1 (0.965)
NetMHCIIpan-4.0 (0.965)
NetMHCIIpan-3.2 (0.896)
NetMHCII-2.3 (0.918)
BERTMHC (0.904)
MHCnuggets (0.789)

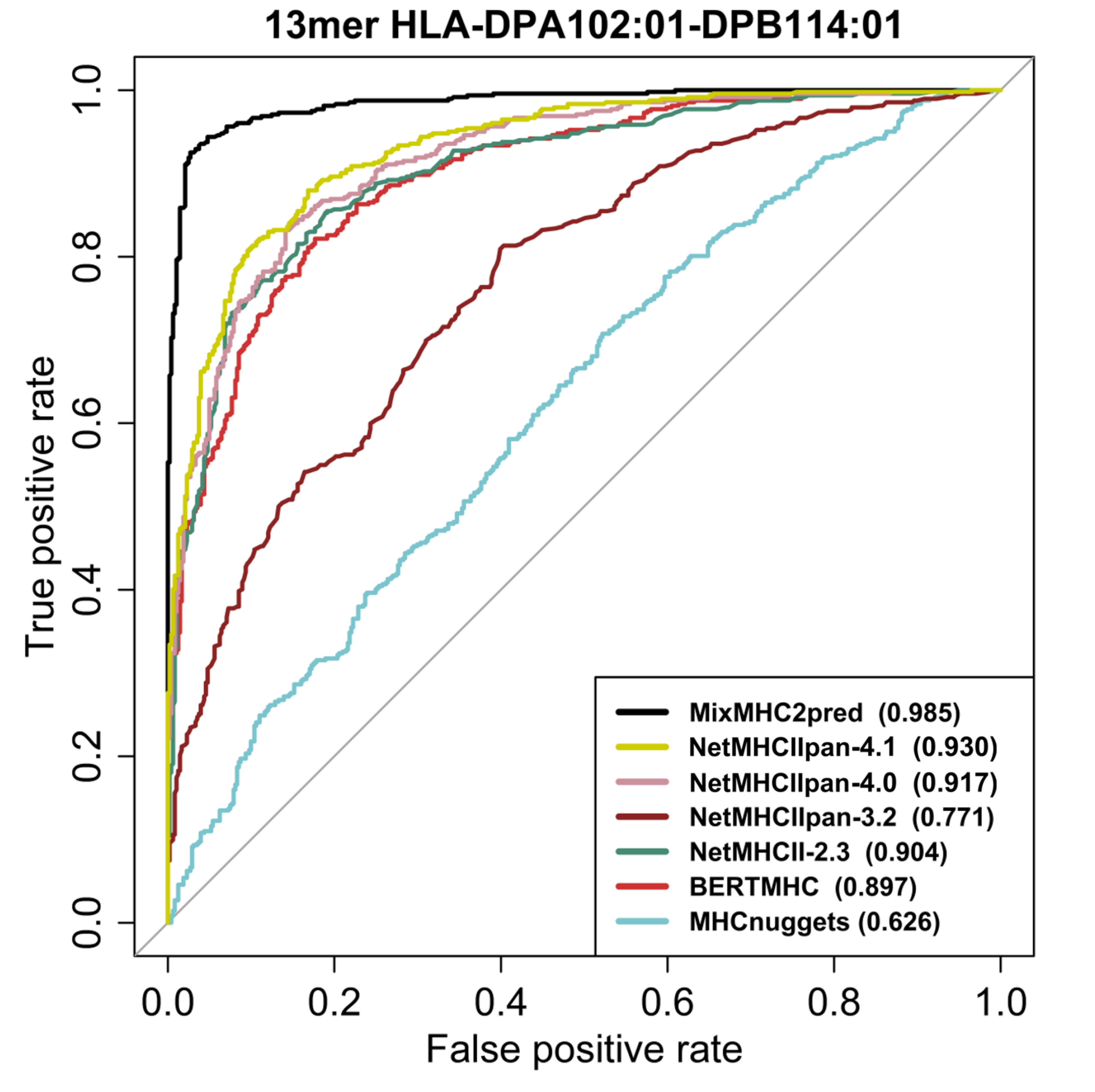

13mer HLA-DPA102:01-DPB114:01
True positive rate
False positive rate
0.0
0.2
0.4
0.6
0.8
1.0
MixMHC2pred (0.985)
NetMHCIIpan-4.1 (0.930)
NetMHCIIpan-4.0 (0.917)
NetMHCIIpan-3.2 (0.771)
NetMHCII-2.3 (0.904)
BERTMHC (0.897)
MHCnuggets (0.626)

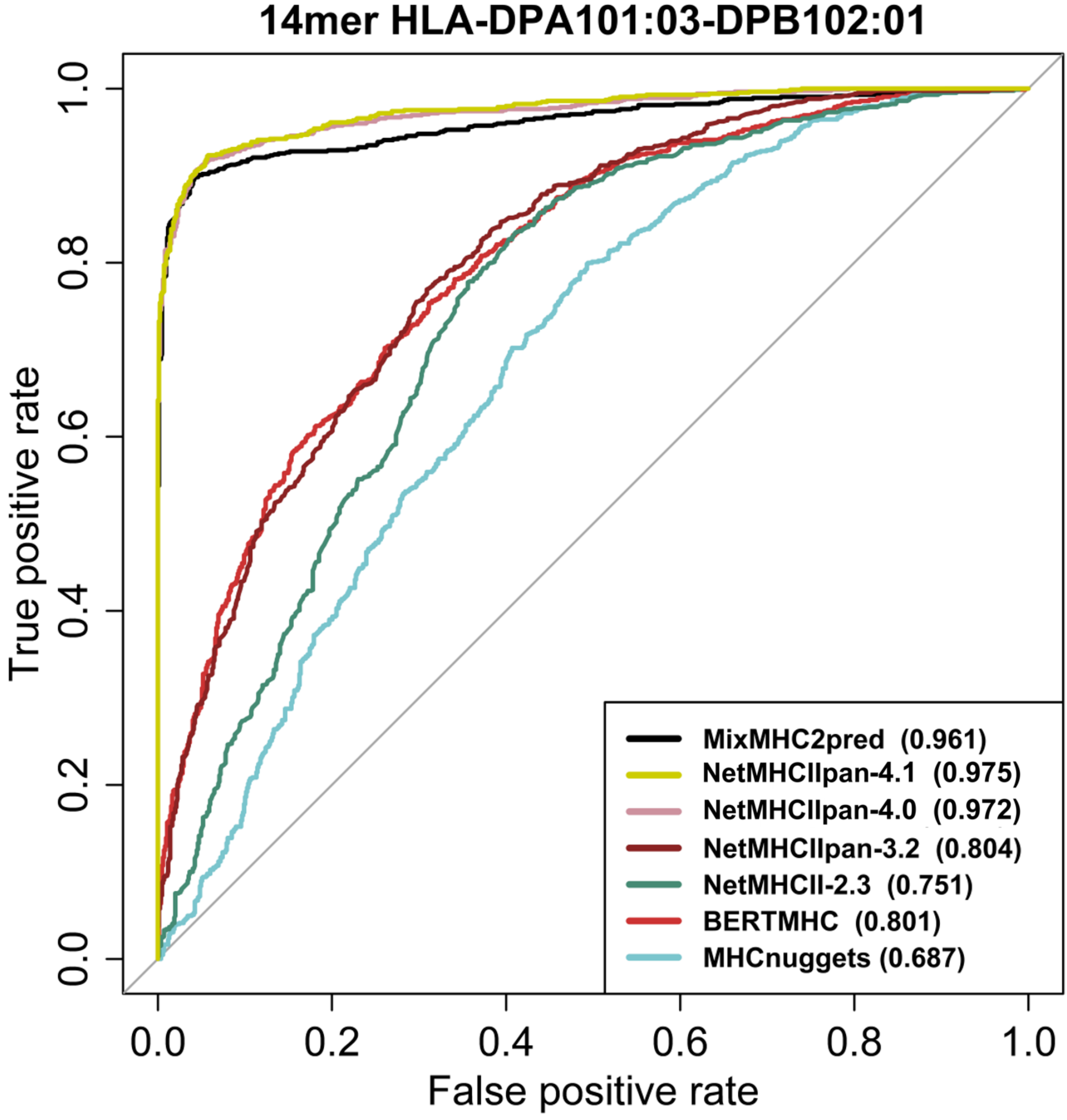
14mer HLA-DPA101:03-DPB102:01
True positive rate
False positive rate
0.0
0.2
0.4
0.6
0.8
1.0
MixMHC2pred (0.961)
NetMHCIIpan-4.1 (0.975)
NetMHCIIpan-4.0 (0.972)
NetMHCIIpan-3.2 (0.804)
NetMHCII-2.3 (0.751)
BERTMHC (0.801)
MHCnuggets (0.687)

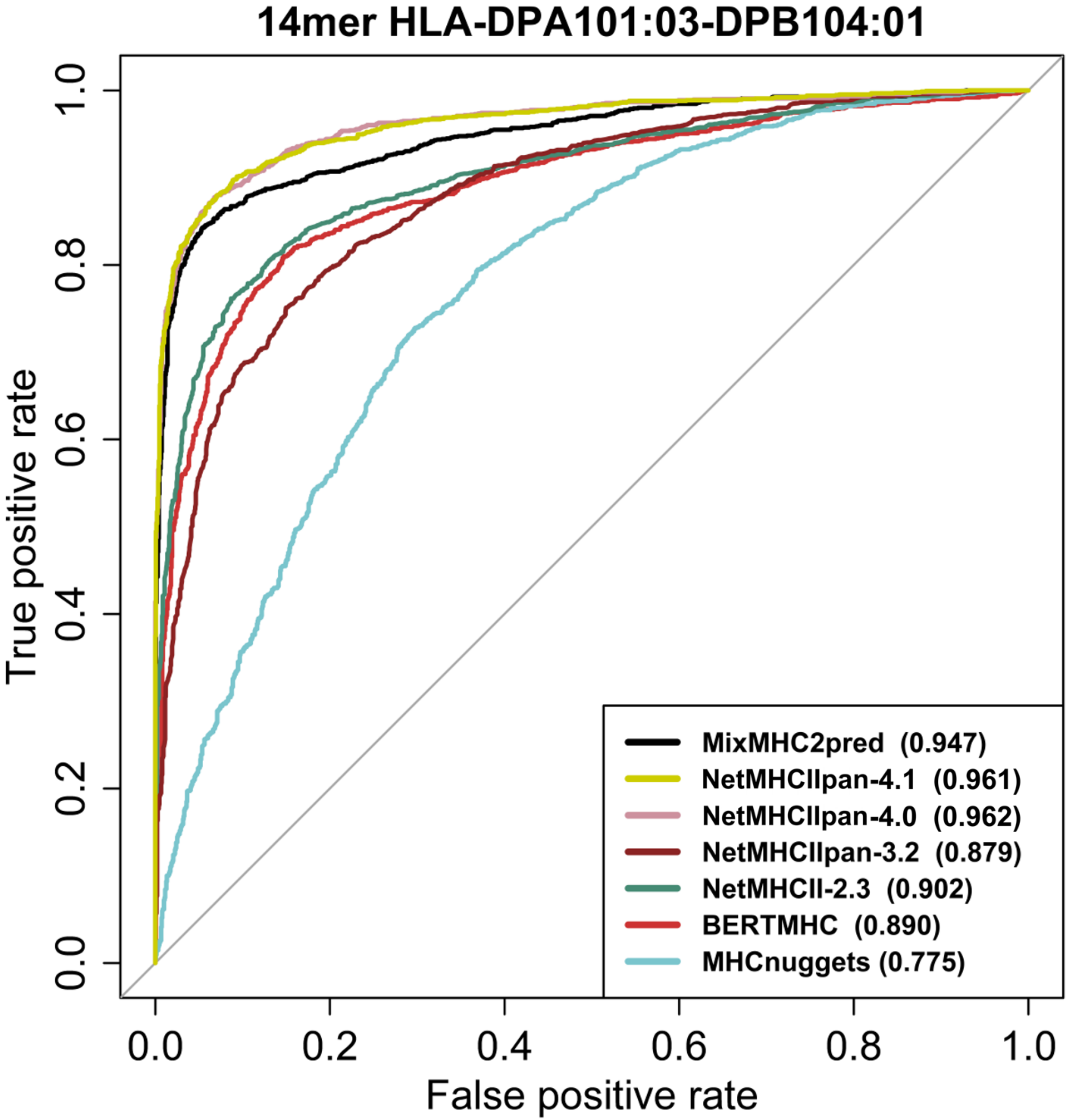

14mer HLA-DPA101:03-DPB104:01
True positive rate
False positive rate
0.0
0.2
0.4
0.6
0.8
1.0
MixMHC2pred (0.947)
NetMHCIIpan-4.1 (0.961)
NetMHCIIpan-4.0 (0.962)
NetMHCIIpan-3.2 (0.879)
NetMHCII-2.3 (0.902)
BERTMHC (0.890)
MHCnuggets (0.775)

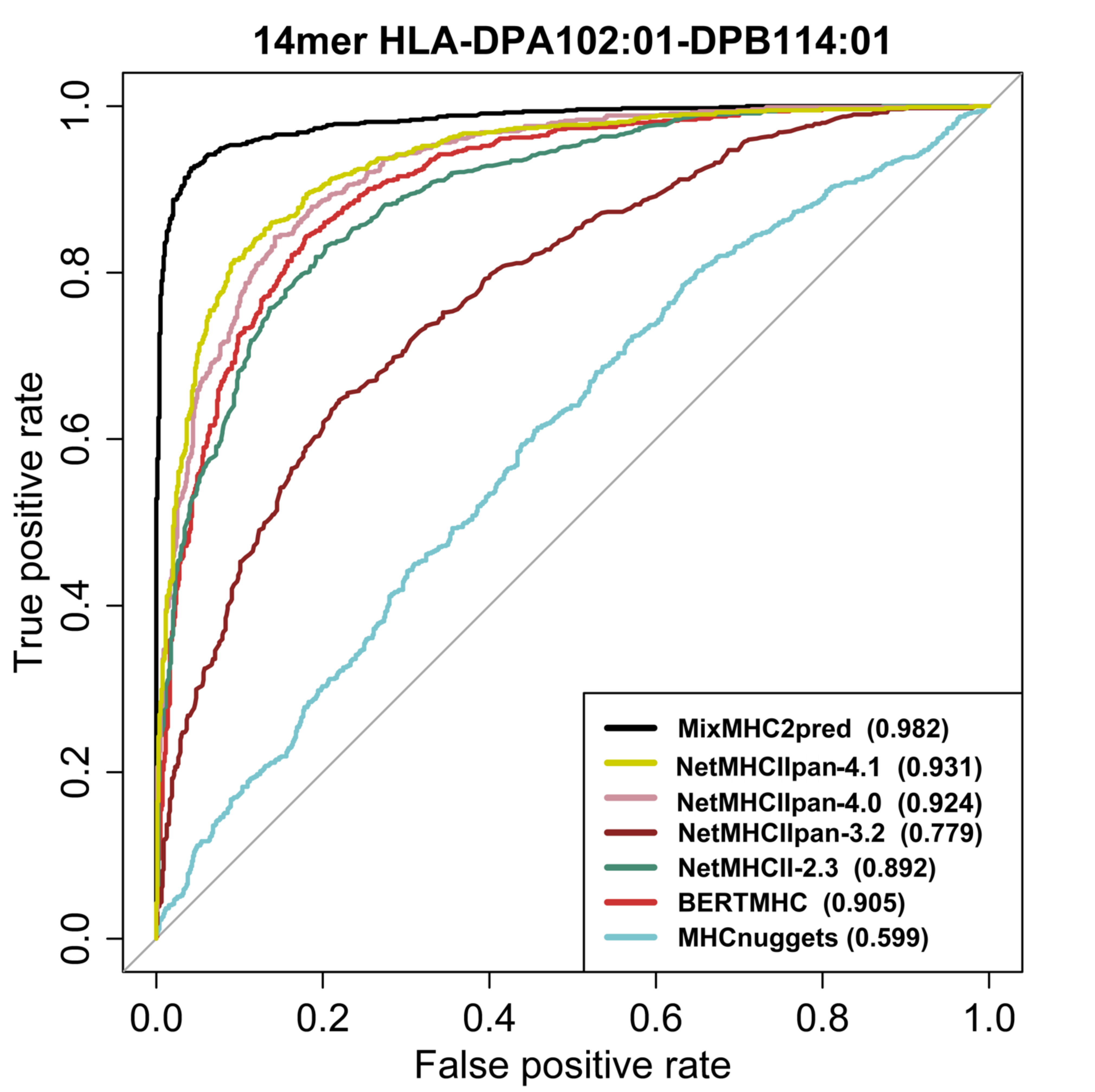
14mer HLA-DPA102:01-DPB114:01
True positive rate
False positive rate
0.0
0.2
0.4
0.6
0.8
1.0
MixMHC2pred (0.982)
NetMHCIIpan-4.1 (0.931)
NetMHCIIpan-4.0 (0.924)
NetMHCIIpan-3.2 (0.779)
NetMHCII-2.3 (0.892)
BERTMHC (0.905)
MHCnuggets (0.599)

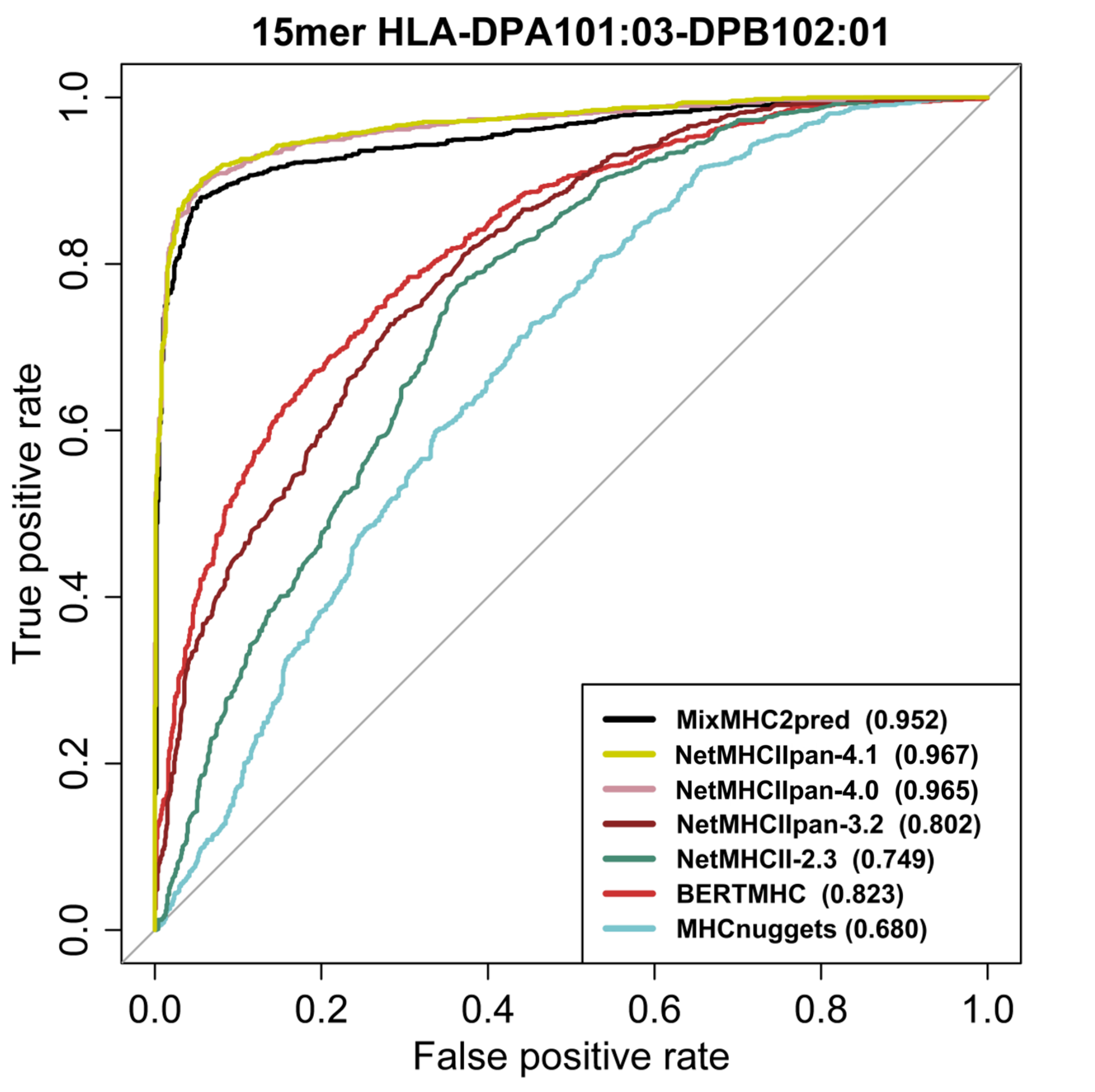

15mer HLA-DPA101:03-DPB102:01
True positive rate
False positive rate
0.0
0.2
0.4
0.6
0.8
1.0
MixMHC2pred (0.952)
NetMHCIIpan-4.1 (0.967)
NetMHCIIpan-4.0 (0.965)
NetMHCIIpan-3.2 (0.802)
NetMHCII-2.3 (0.749)
BERTMHC (0.823)
MHCnuggets (0.680)

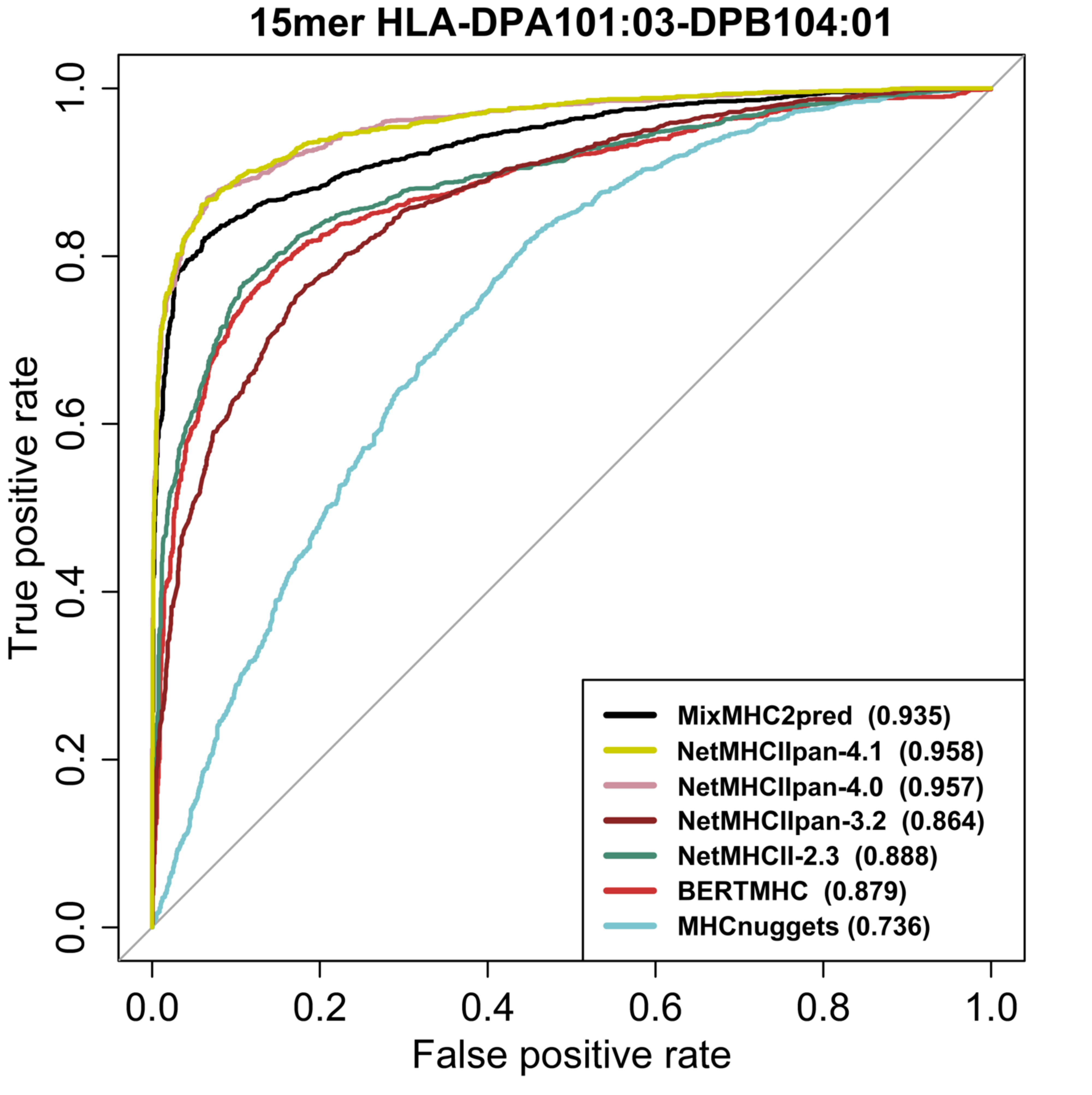
15mer HLA-DPA101:03-DPB104:01
True positive rate
False positive rate
0.0
0.2
0.4
0.6
0.8
1.0
MixMHC2pred (0.935)
NetMHCIIpan-4.1 (0.958)
NetMHCIIpan-4.0 (0.957)
NetMHCIIpan-3.2 (0.864)
NetMHCII-2.3 (0.888)
BERTMHC (0.879)
MHCnuggets (0.736)

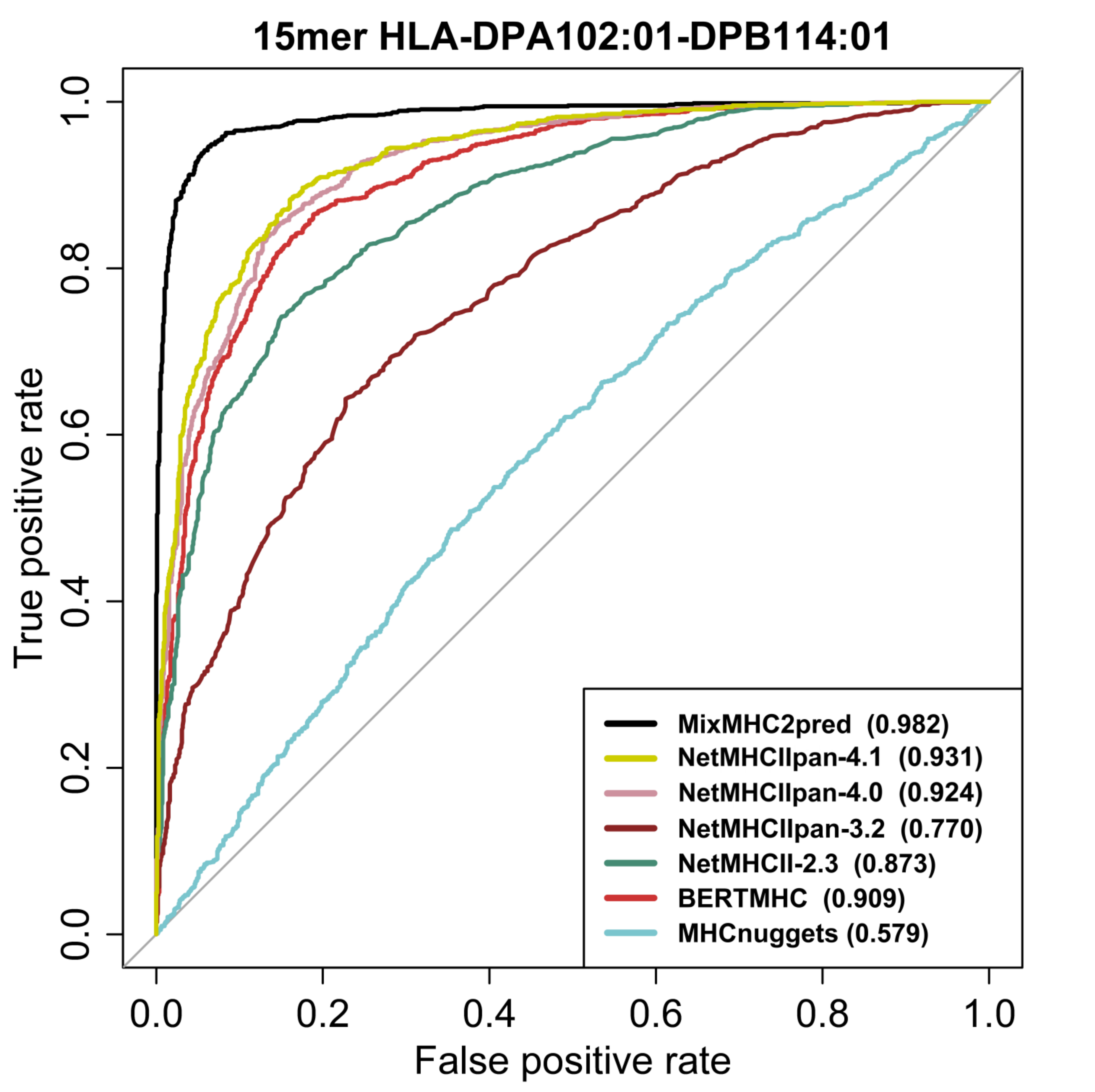

15mer HLA-DPA102:01-DPB114:01
True positive rate
False positive rate
0.0
0.2
0.4
0.6
0.8
1.0
MixMHC2pred (0.982)
NetMHCIIpan-4.1 (0.931)
NetMHCIIpan-4.0 (0.924)
NetMHCIIpan-3.2 (0.770)
NetMHCII-2.3 (0.873)
BERTMHC (0.909)
MHCnuggets (0.579)

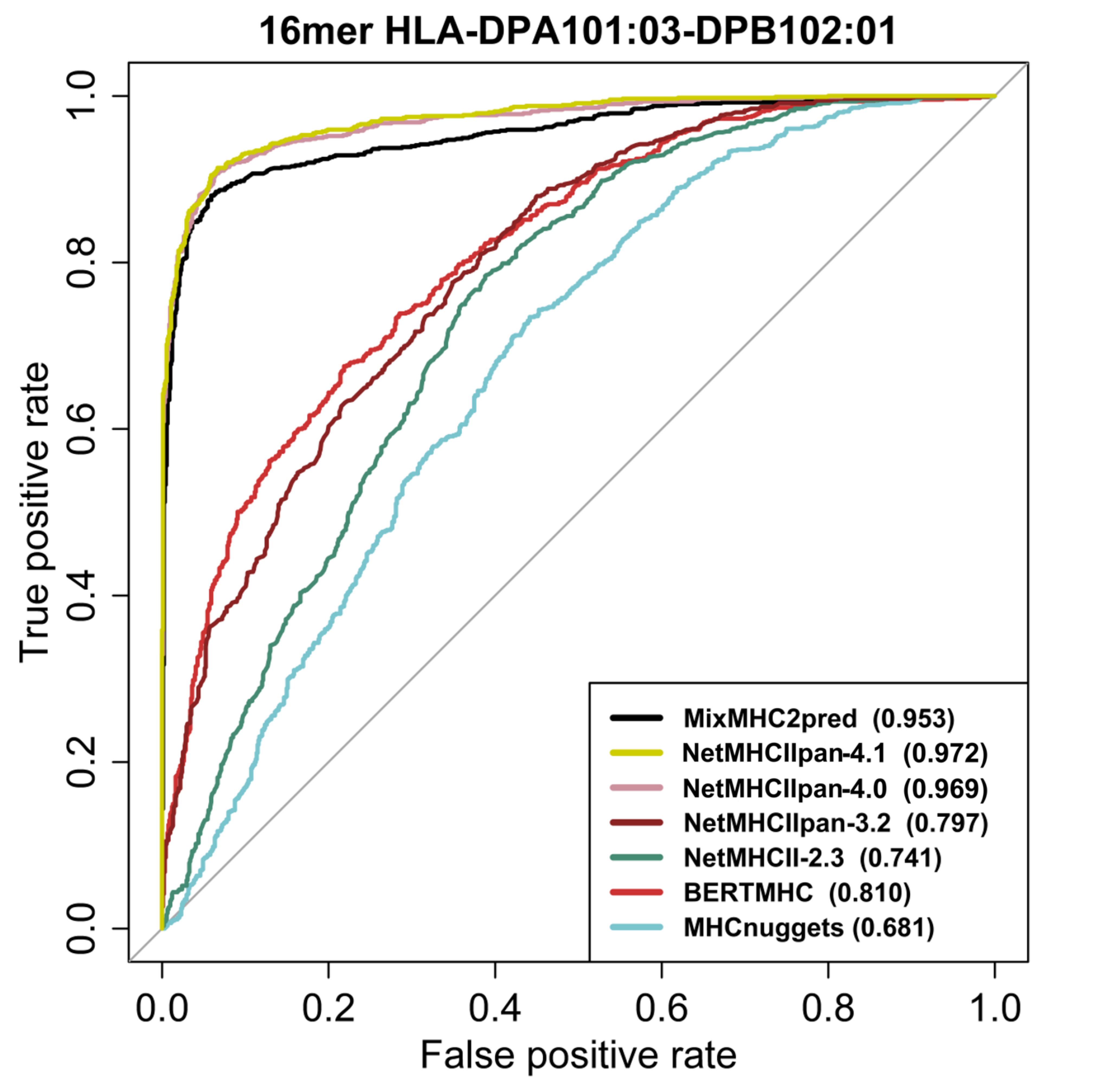
16mer HLA-DPA101:03-DPB102:01
True positive rate
False positive rate
0.0
0.2
0.4
0.6
0.8
1.0
MixMHC2pred (0.953)
NetMHCIIpan-4.1 (0.972)
NetMHCIIpan-4.0 (0.969)
NetMHCIIpan-3.2 (0.797)
NetMHCII-2.3 (0.741)
BERTMHC (0.810)
MHCnuggets (0.681)

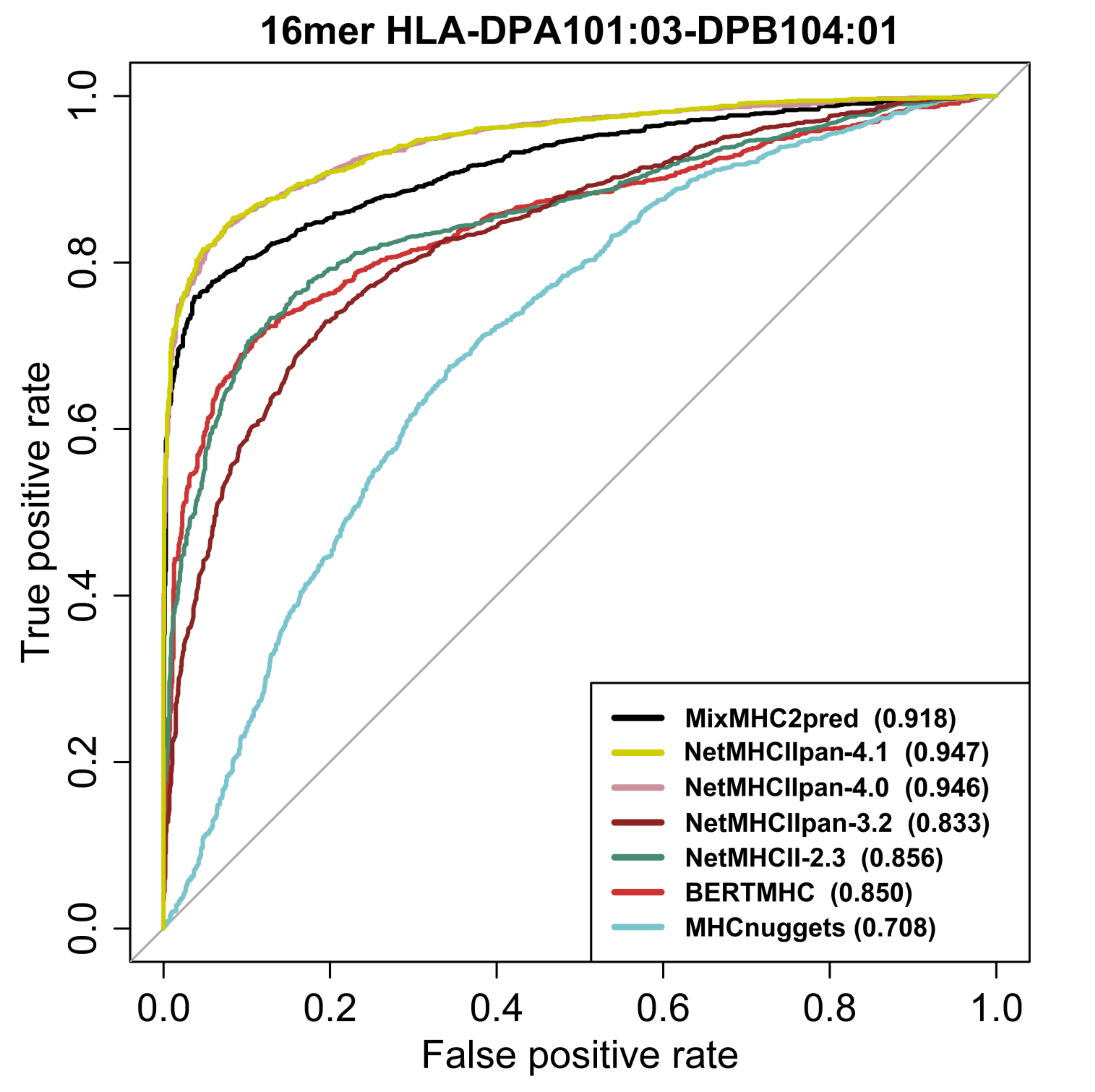
16mer HLA-DPA101:03-DPB104:01
True positive rate
False positive rate
0.0
0.2
0.4
0.6
0.8
1.0
MixMHC2pred (0.918)
NetMHCIIpan-4.1 (0.947)
NetMHCIIpan-4.0 (0.946)
NetMHCIIpan-3.2 (0.833)
NetMHCII-2.3 (0.856)
BERTMHC (0.850)
MHCnuggets (0.708)

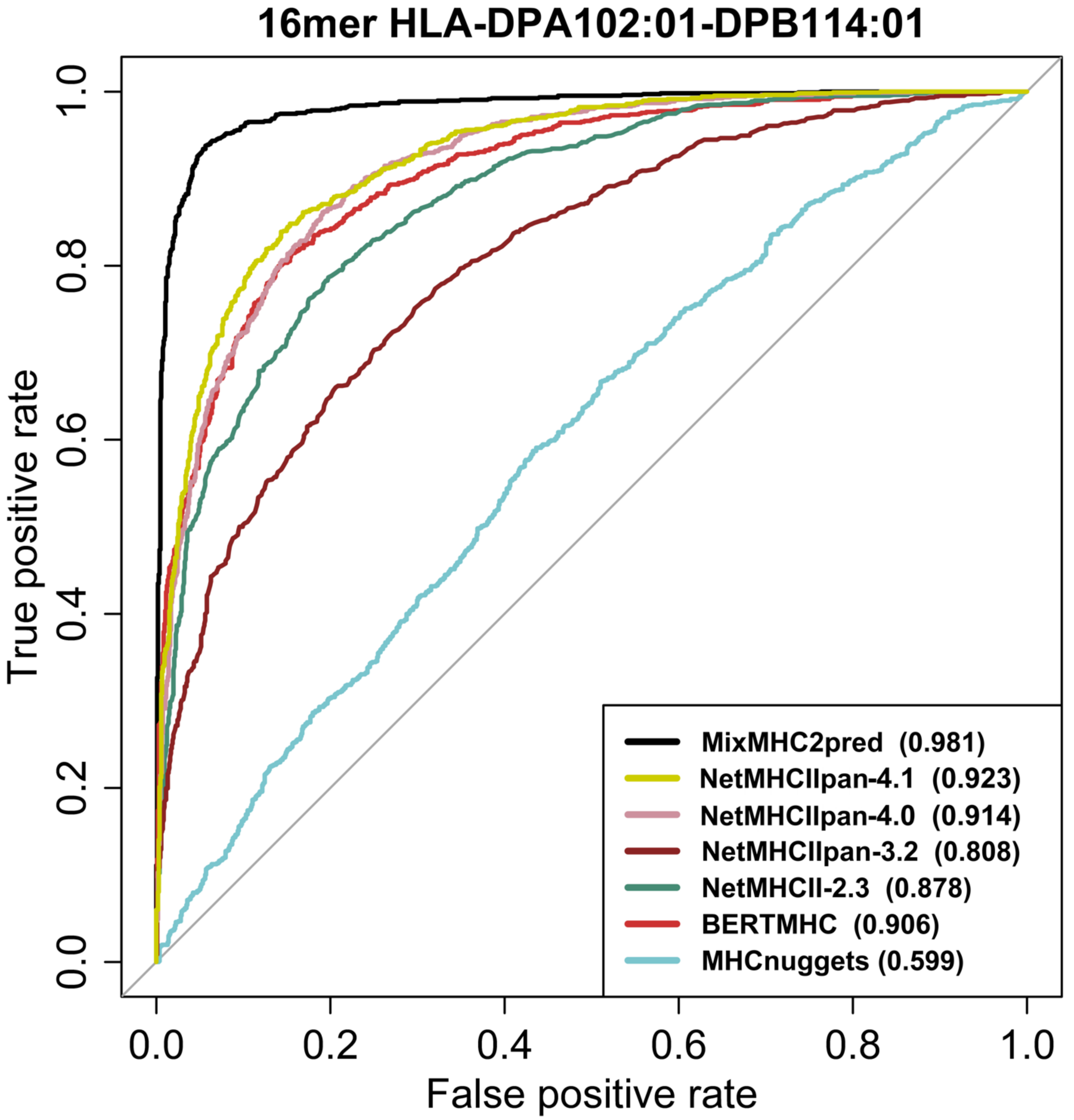
16mer HLA-DPA102:01-DPB114:01
True positive rate
False positive rate
0.0
0.2
0.4
0.6
0.8
1.0
MixMHC2pred (0.981)
NetMHCIIpan-4.1 (0.923)
NetMHCIIpan-4.0 (0.914)
NetMHCIIpan-3.2 (0.808)
NetMHCII-2.3 (0.878)
BERTMHC (0.906)
MHCnuggets (0.599)

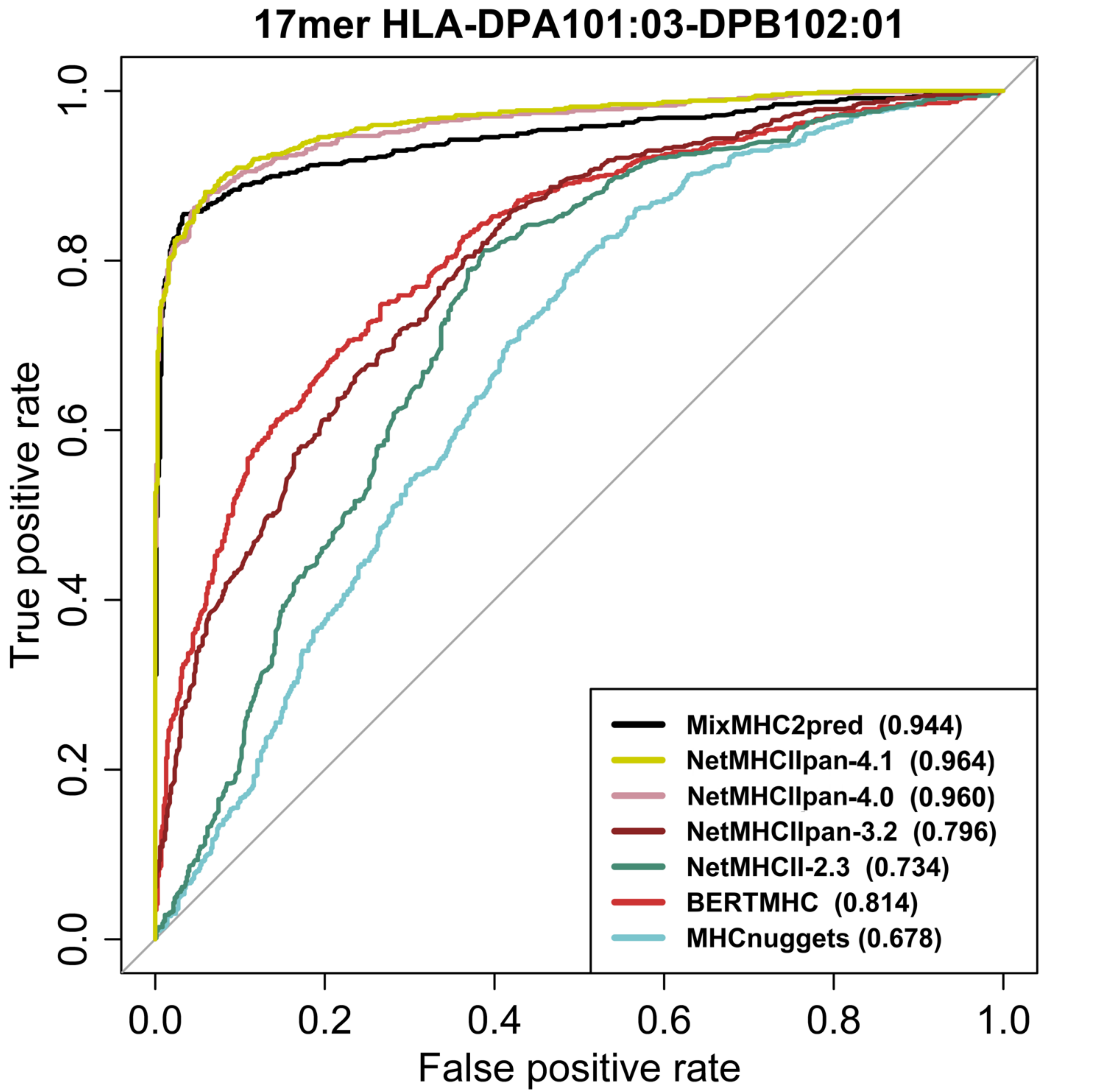
17mer HLA-DPA101:03-DPB102:01
True positive rate
False positive rate
0.0
0.2
0.4
0.6
0.8
1.0
MixMHC2pred (0.944)
NetMHCIIpan-4.1 (0.964)
NetMHCIIpan-4.0 (0.960)
NetMHCIIpan-3.2 (0.796)
NetMHCII-2.3 (0.734)
BERTMHC (0.814)
MHCnuggets (0.678)

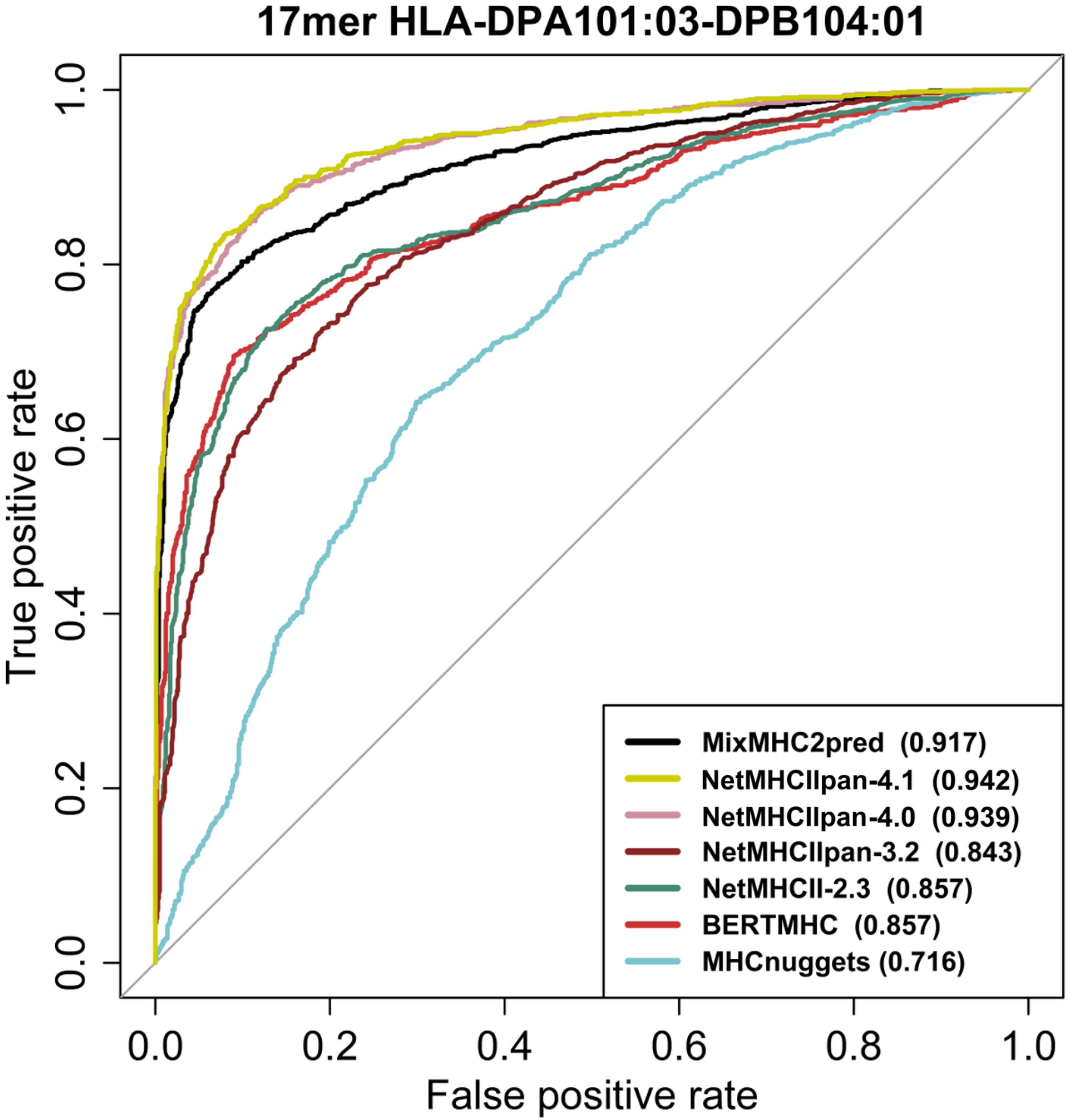

17mer HLA-DPA101:03-DPB104:01
True positive rate
False positive rate
0.0
0.2
0.4
0.6
0.8
1.0
MixMHC2pred (0.917)
NetMHCIIpan-4.1 (0.942)
NetMHCIIpan-4.0 (0.939)
NetMHCIIpan-3.2 (0.843)
NetMHCII-2.3 (0.857)
BERTMHC (0.857)
MHCnuggets (0.716)

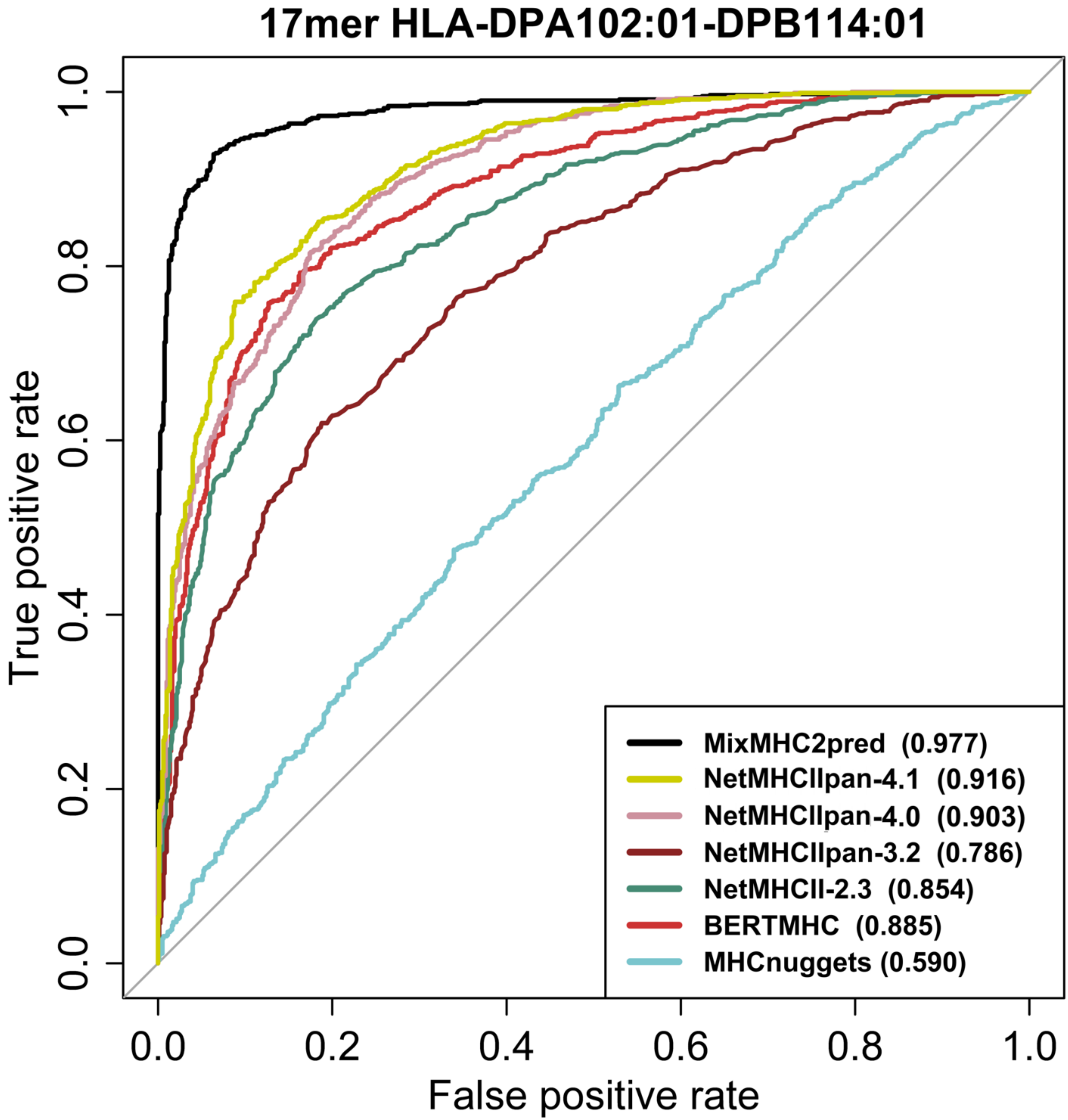
17mer HLA-DPA102:01-DPB114:01
True positive rate
False positive rate
0.0
0.2
0.4
0.6
0.8
1.0
MixMHC2pred (0.977)
NetMHCIIpan-4.1 (0.916)
NetMHCIIpan-4.0 (0.903)
NetMHCIIpan-3.2 (0.786)
NetMHCII-2.3 (0.854)
BERTMHC (0.885)
MHCnuggets (0.590)

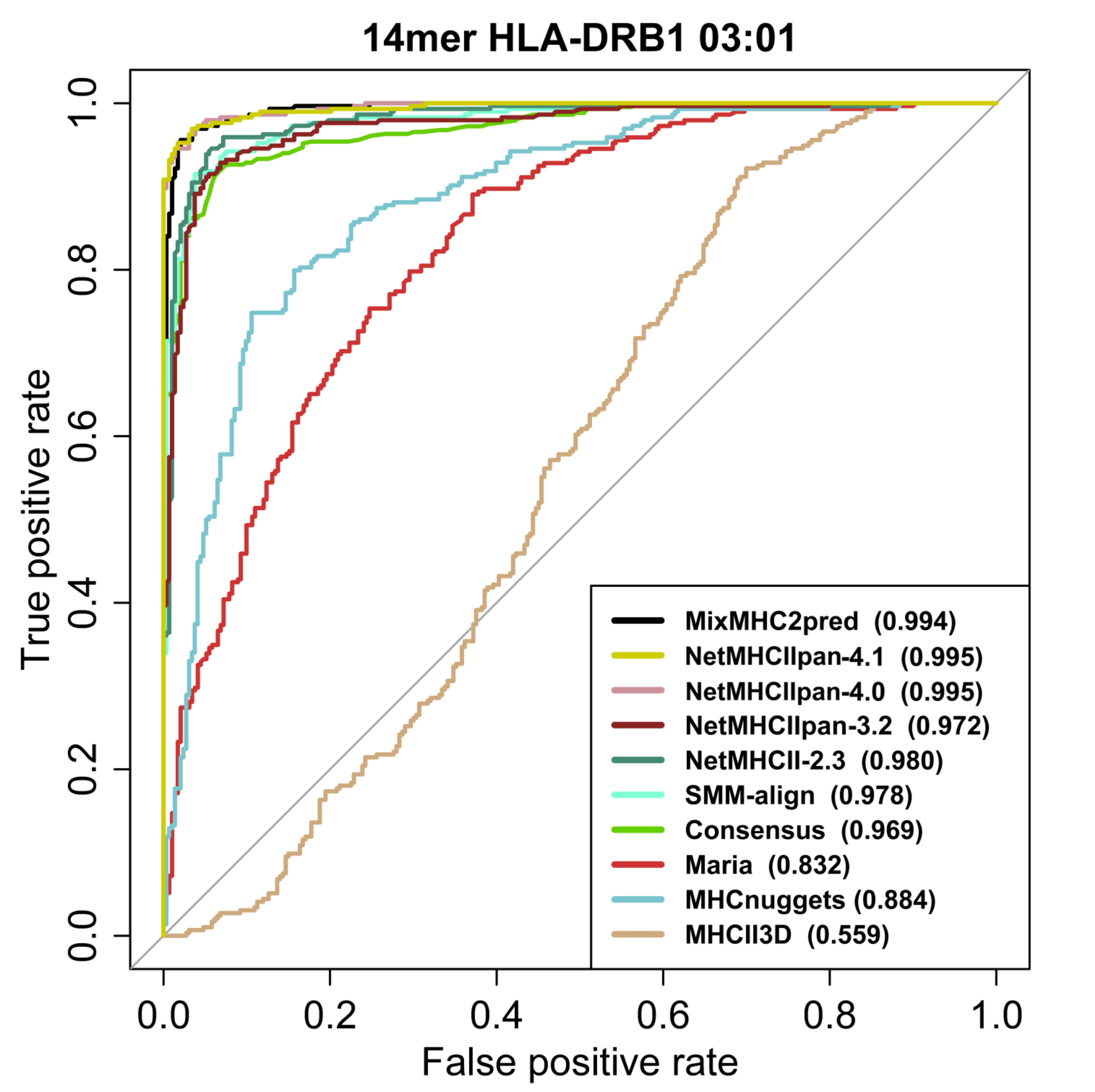
14mer HLA-DRB1 03:01
True positive rate
False positive rate
0.0
0.2
0.4
0.6
0.8
1.0
MixMHC2pred (0.994)
NetMHCIIpan-4.1 (0.995)
NetMHCIIpan-4.0 (0.995)
NetMHCIIpan-3.2 (0.972)
NetMHCII-2.3 (0.980)
SMM-align (0.978)
Consensus (0.969)
Maria (0.832)
MHCnuggets (0.884)
MHCII3D (0.559)

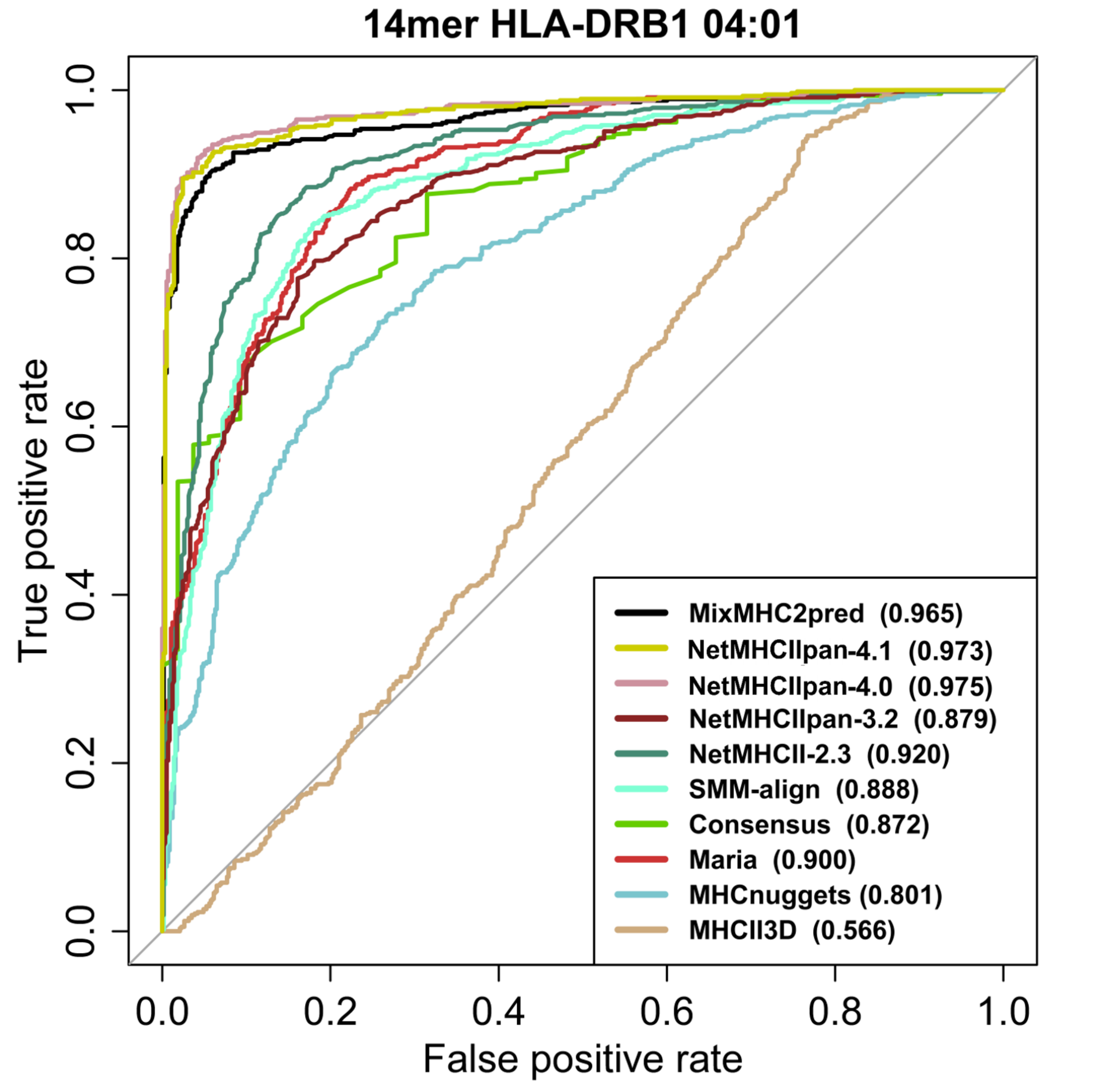
14mer HLA-DRB1 04:01
True positive rate
False positive rate
0.0
0.2
0.4
0.6
0.8
1.0
MixMHC2pred (0.965)
NetMHCIIpan-4.1 (0.973)
NetMHCIIpan-4.0 (0.975)
NetMHCIIpan-3.2 (0.879)
NetMHCII-2.3 (0.920)
SMM-align (0.888)
Consensus (0.872)
Maria (0.900)
MHCnuggets (0.801)
MHCII3D (0.566)

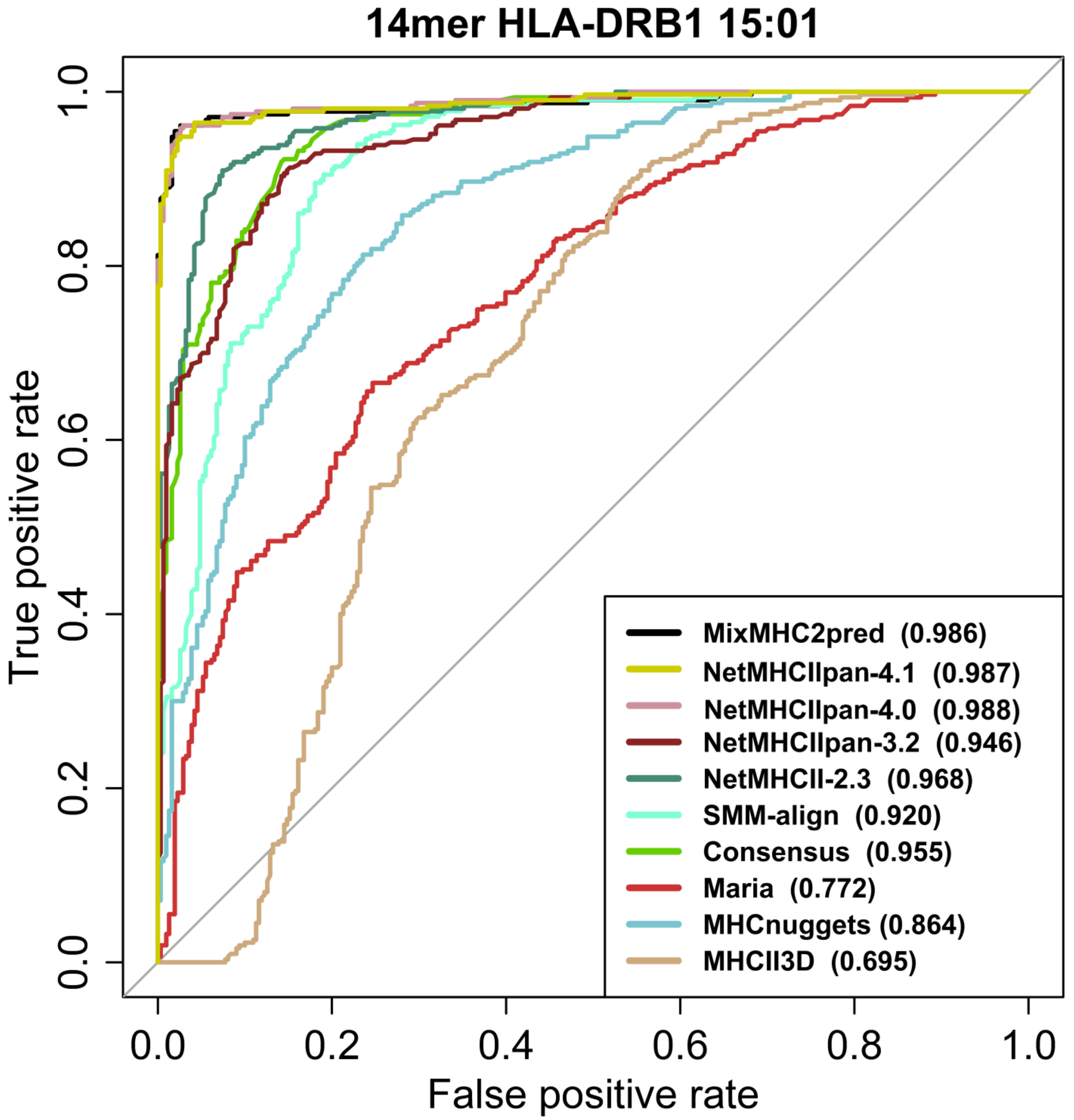
14mer HLA-DRB1 15:01
True positive rate
False positive rate
0.0
0.2
0.4
0.6
0.8
1.0
MixMHC2pred (0.986)
NetMHCIIpan-4.1 (0.987)
NetMHCIIpan-4.0 (0.988)
NetMHCIIpan-3.2 (0.946)
NetMHCII-2.3 (0.968)
SMM-align (0.920)
Consensus (0.955)
Maria (0.772)
MHCnuggets (0.864)
MHCII3D (0.695)

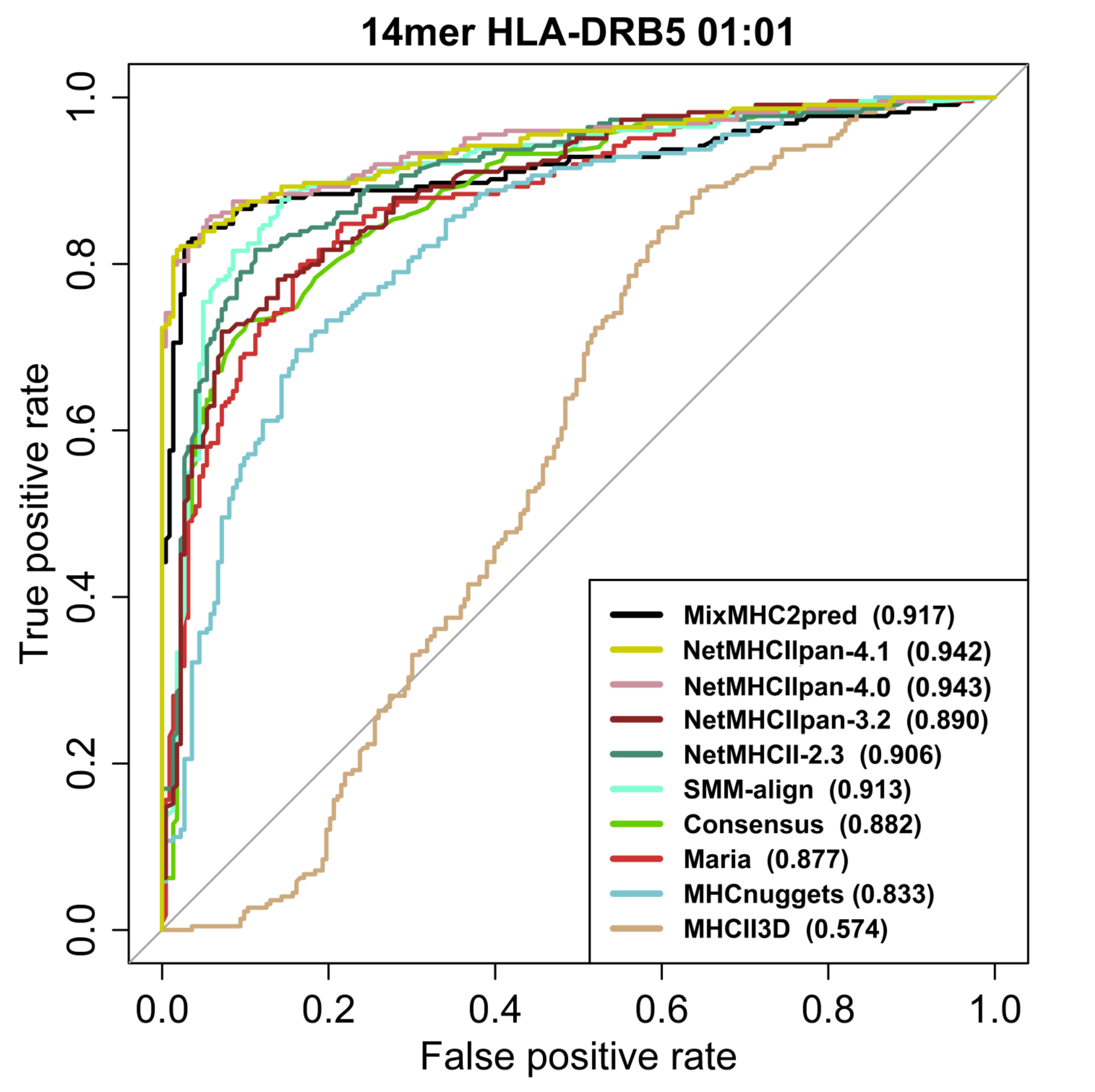
14mer HLA-DRB5 01:01
True positive rate
False positive rate
0.0
0.2
0.4
0.6
0.8
1.0
MixMHC2pred (0.917)
NetMHCIIpan-4.1 (0.942)
NetMHCIIpan-4.0 (0.943)
NetMHCIIpan-3.2 (0.890)
NetMHCII-2.3 (0.906)
SMM-align (0.913)
Consensus (0.882)
Maria (0.877)
MHCnuggets (0.833)
MHCII3D (0.574)

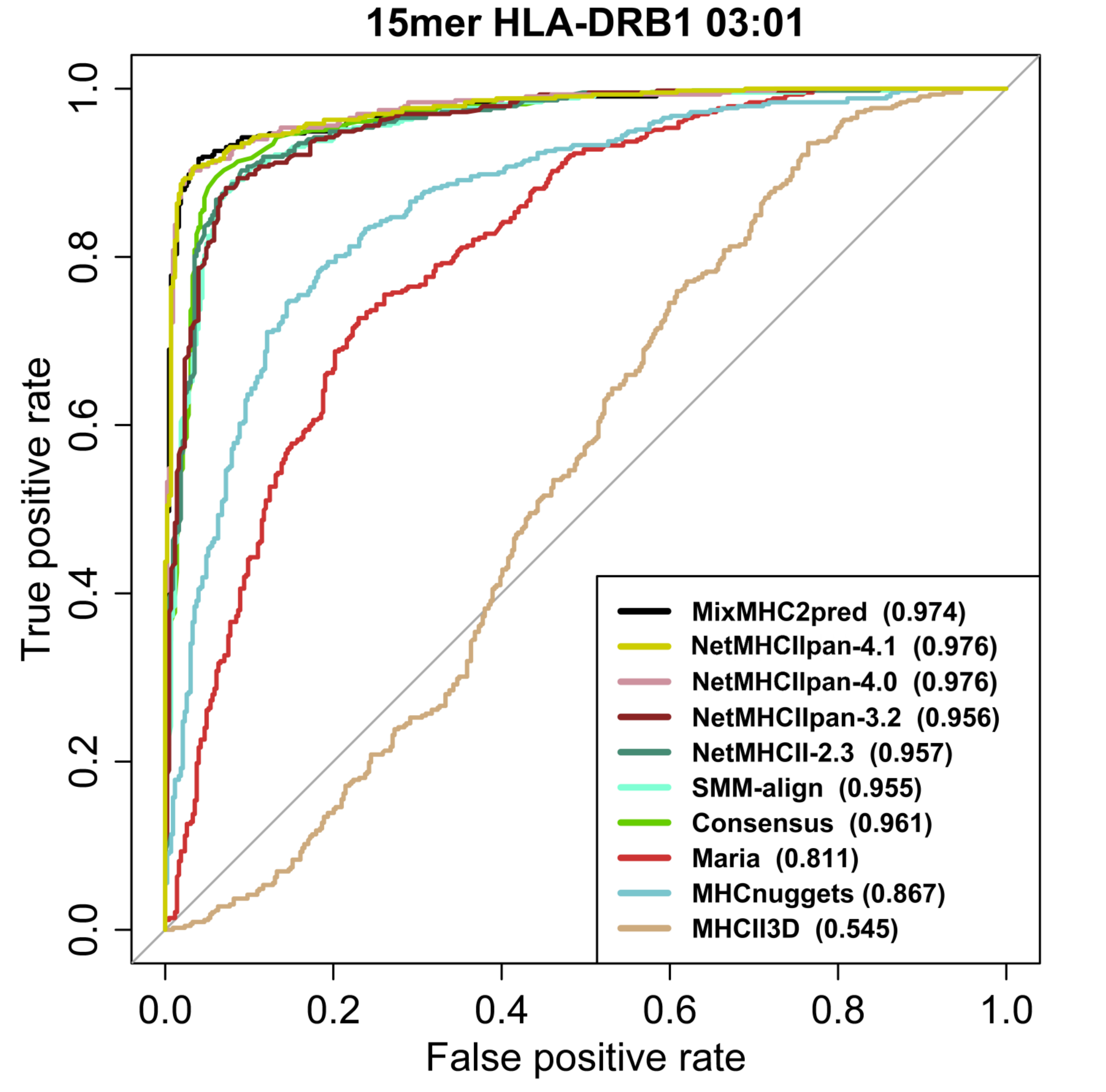
15mer HLA-DRB1 03:01
True positive rate
False positive rate
0.0
0.2
0.4
0.6
0.8
1.0
MixMHC2pred (0.974)
NetMHCIIpan-4.1 (0.976)
NetMHCIIpan-4.0 (0.976)
NetMHCIIpan-3.2 (0.956)
NetMHCII-2.3 (0.957)
SMM-align (0.955)
Consensus (0.961)
Maria (0.811)
MHCnuggets (0.867)
MHCII3D (0.545)

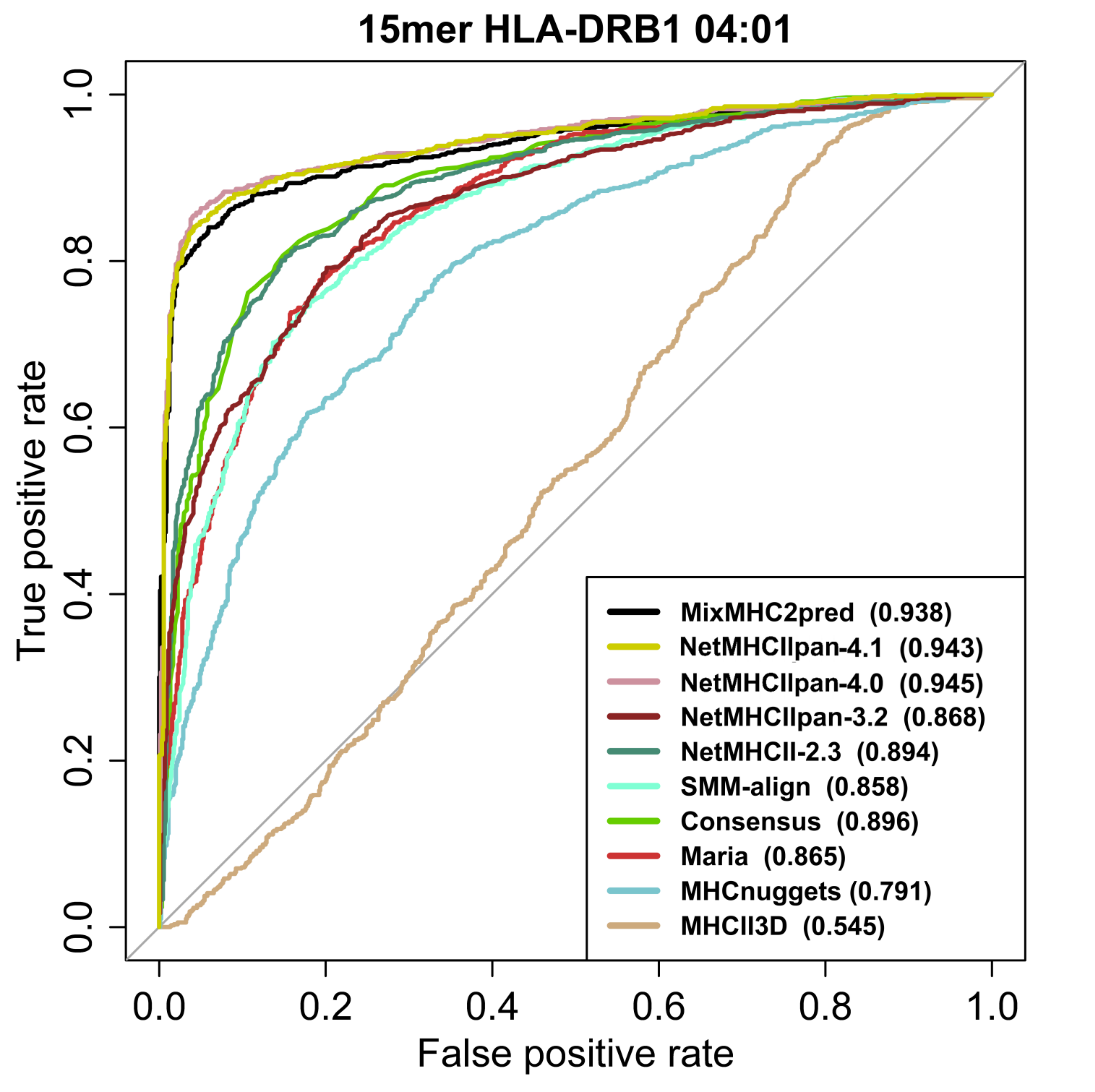
15mer HLA-DRB1 04:01
True positive rate
False positive rate
0.0
0.2
0.4
0.6
0.8
1.0
MixMHC2pred (0.938)
NetMHCIIpan-4.1 (0.943)
NetMHCIIpan-4.0 (0.945)
NetMHCIIpan-3.2 (0.868)
NetMHCII-2.3 (0.894)
SMM-align (0.858)
Consensus (0.896)
Maria (0.865)
MHCnuggets (0.791)
MHCII3D (0.545)

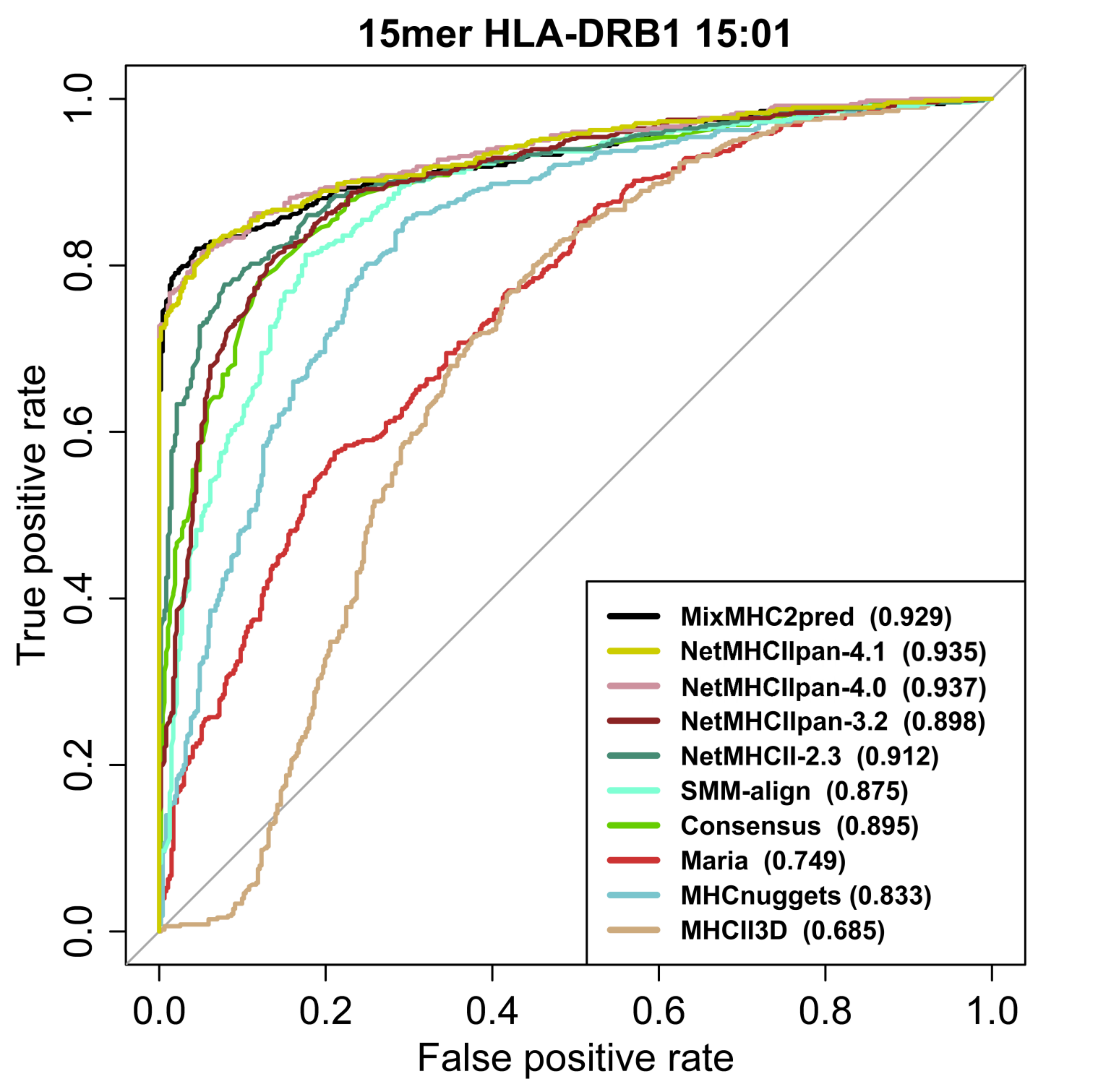

15mer HLA-DRB1 15:01
True positive rate
False positive rate
0.0
0.2
0.4
0.6
0.8
1.0
MixMHC2pred (0.929)
NetMHCIIpan-4.1 (0.935)
NetMHCIIpan-4.0 (0.937)
NetMHCIIpan-3.2 (0.898)
NetMHCII-2.3 (0.912)
SMM-align (0.875)
Consensus (0.895)
Maria (0.749)
MHCnuggets (0.833)
MHCII3D (0.685)

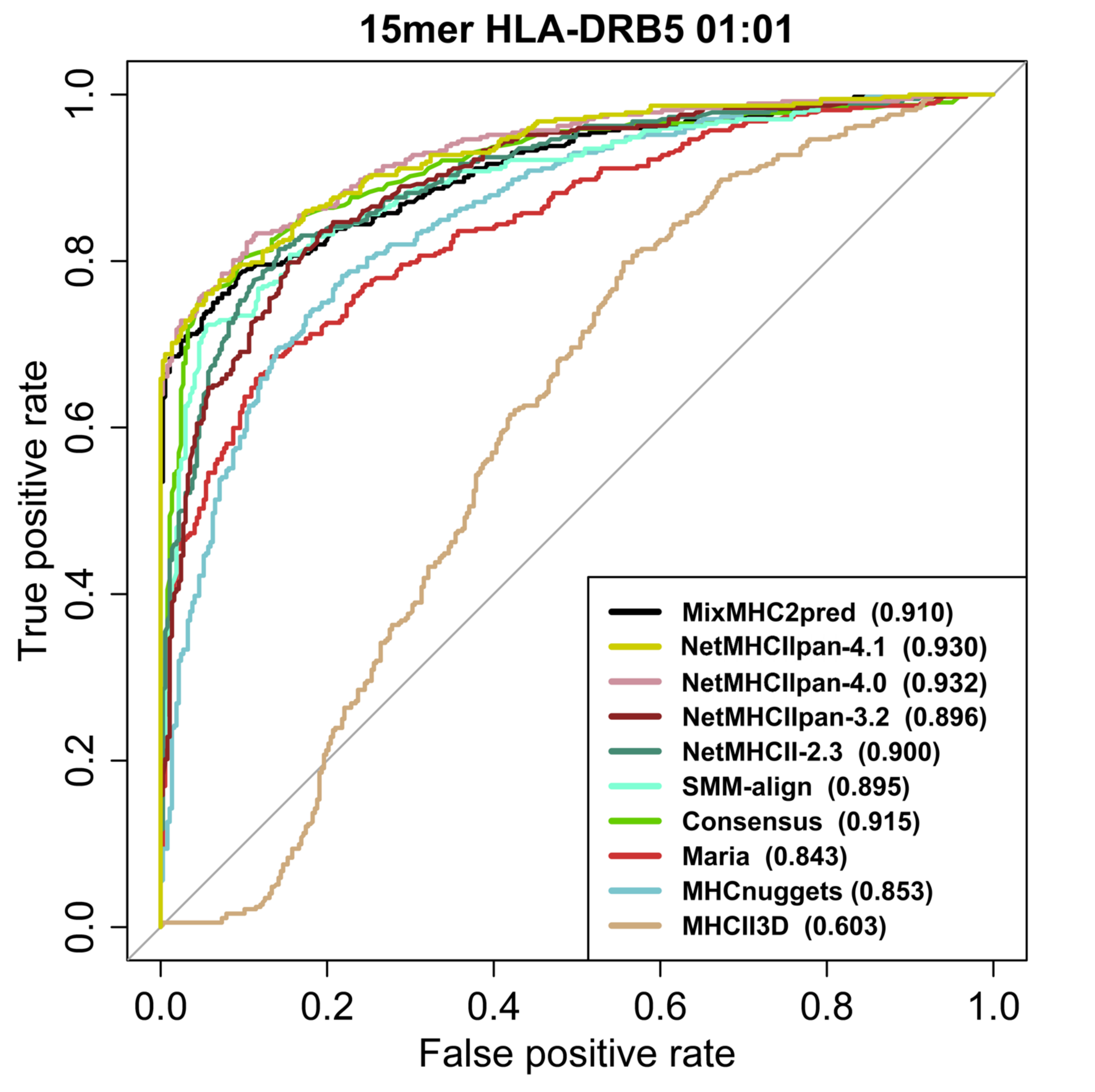

15mer HLA-DRB5 01:01
True positive rate
False positive rate
0.0
0.2
0.4
0.6
0.8
1.0
MixMHC2pred (0.910)
NetMHCIIpan-4.1 (0.930)
NetMHCIIpan-4.0 (0.932)
NetMHCIIpan-3.2 (0.896)
NetMHCII-2.3 (0.900)
SMM-align (0.895)
Consensus (0.915)
Maria (0.843)
MHCnuggets (0.853)
MHCII3D (0.603)

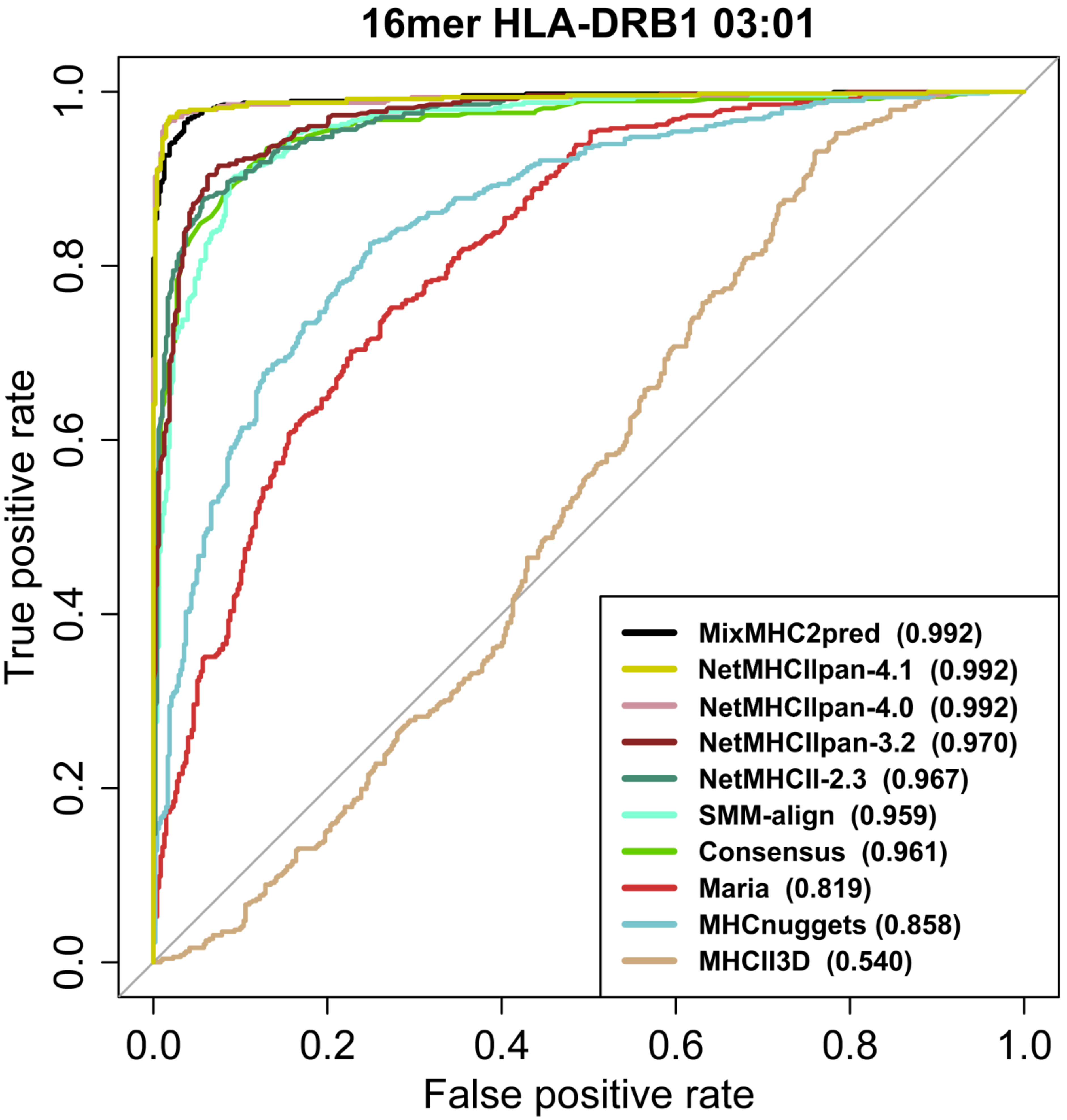
16mer HLA-DRB1 03:01
True positive rate
False positive rate
0.0
0.2
0.4
0.6
0.8
1.0
MixMHC2pred (0.992)
NetMHCIIpan-4.1 (0.992)
NetMHCIIpan-4.0 (0.992)
NetMHCIIpan-3.2 (0.970)
NetMHCII-2.3 (0.967)
SMM-align (0.959)
Consensus (0.961)
Maria (0.819)
MHCnuggets (0.858)
MHCII3D (0.540)

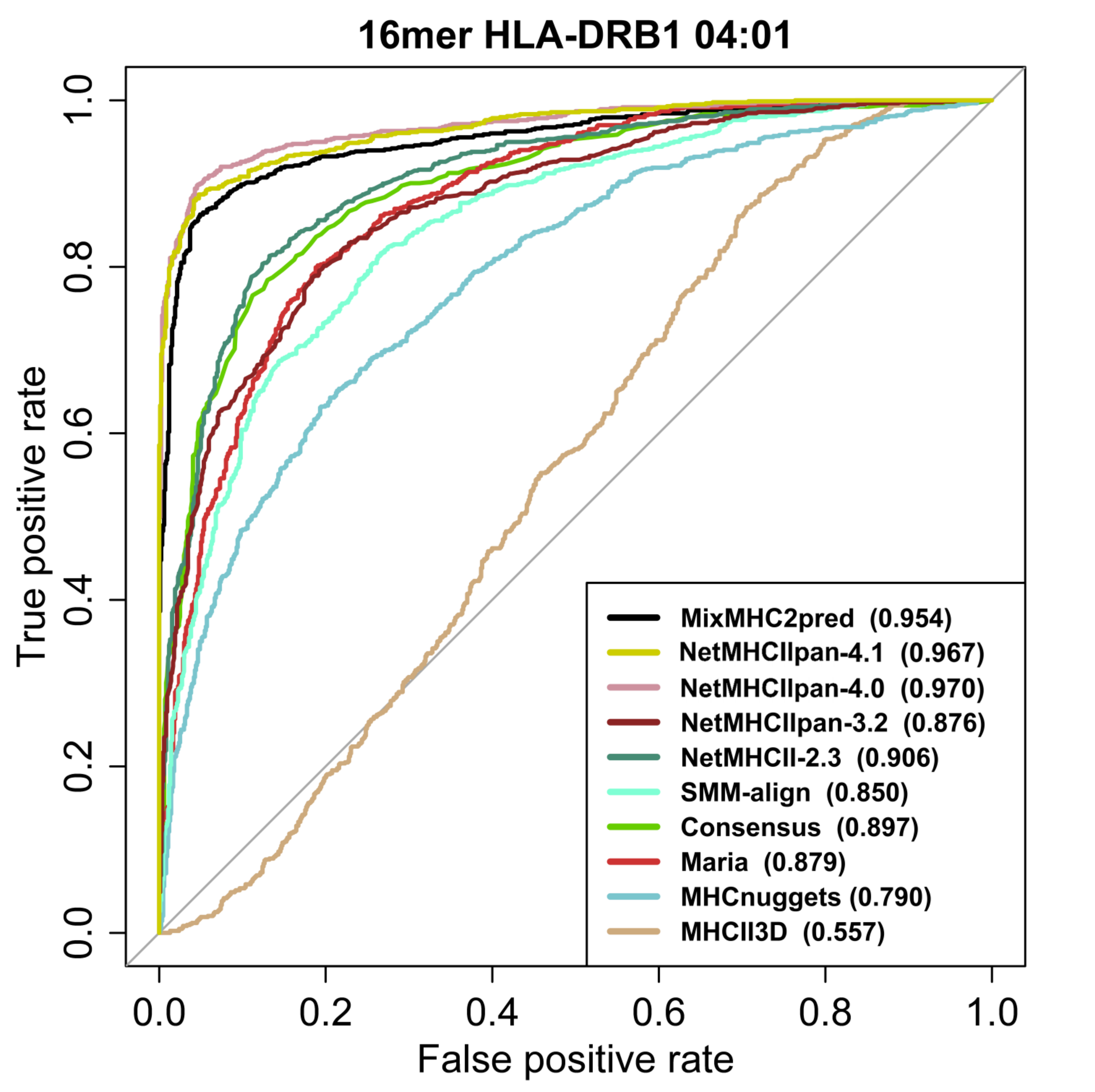

16mer HLA-DRB1 04:01
True positive rate
False positive rate
0.0
0.2
0.4
0.6
0.8
1.0
MixMHC2pred (0.954)
NetMHCIIpan-4.1 (0.967)
NetMHCIIpan-4.0 (0.970)
NetMHCIIpan-3.2 (0.876)
NetMHCII-2.3 (0.906)
SMM-align (0.850)
Consensus (0.897)
Maria (0.879)
MHCnuggets (0.790)
MHCII3D (0.557)

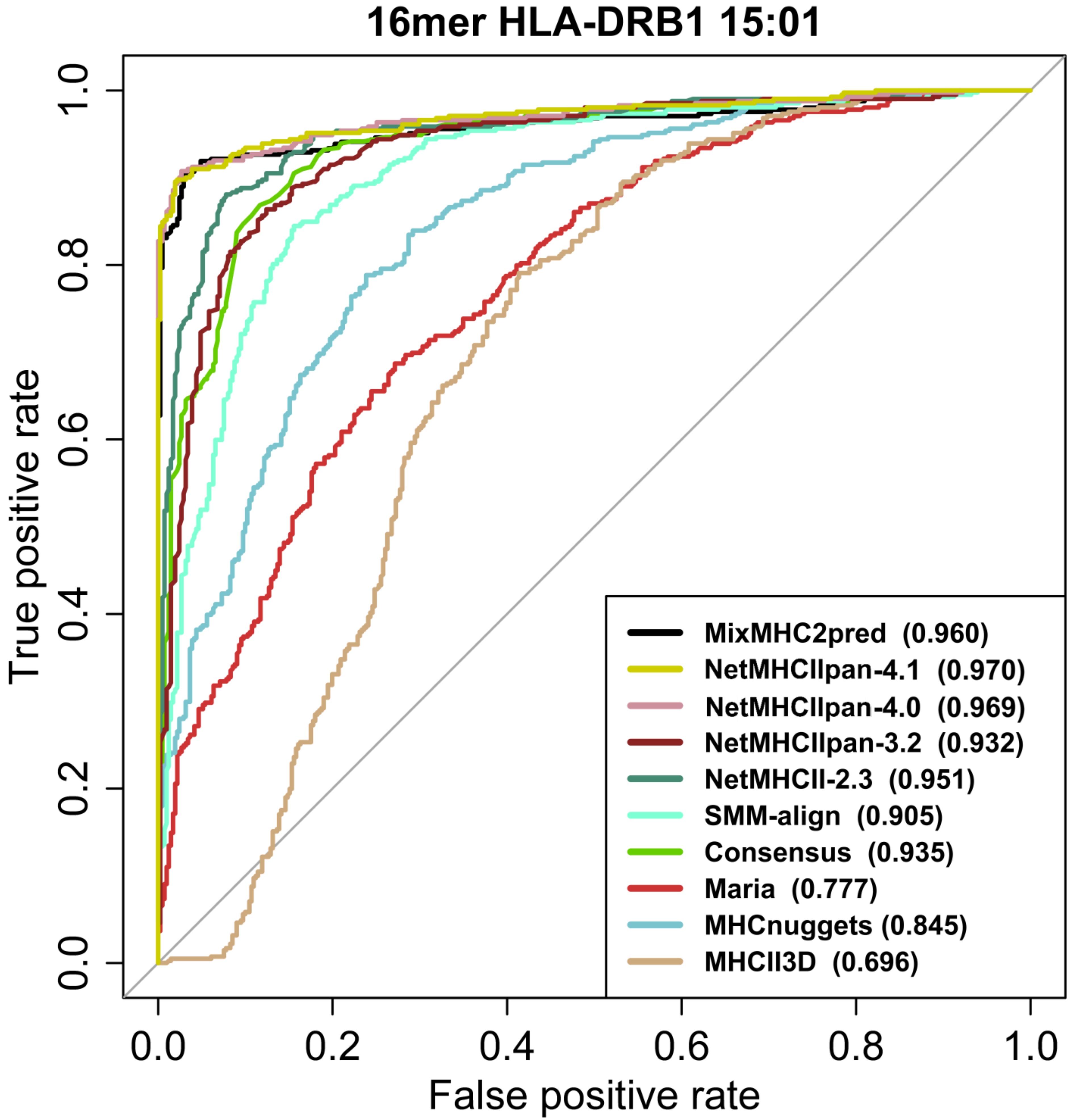
16mer HLA-DRB1 15:01
True positive rate
False positive rate
0.0
0.2
0.4
0.6
0.8
1.0
MixMHC2pred (0.960)
NetMHCIIpan-4.1 (0.970)
NetMHCIIpan-4.0 (0.969)
NetMHCIIpan-3.2 (0.932)
NetMHCII-2.3 (0.951)
SMM-align (0.905)
Consensus (0.935)
Maria (0.777)
MHCnuggets (0.845)
MHCII3D (0.696)

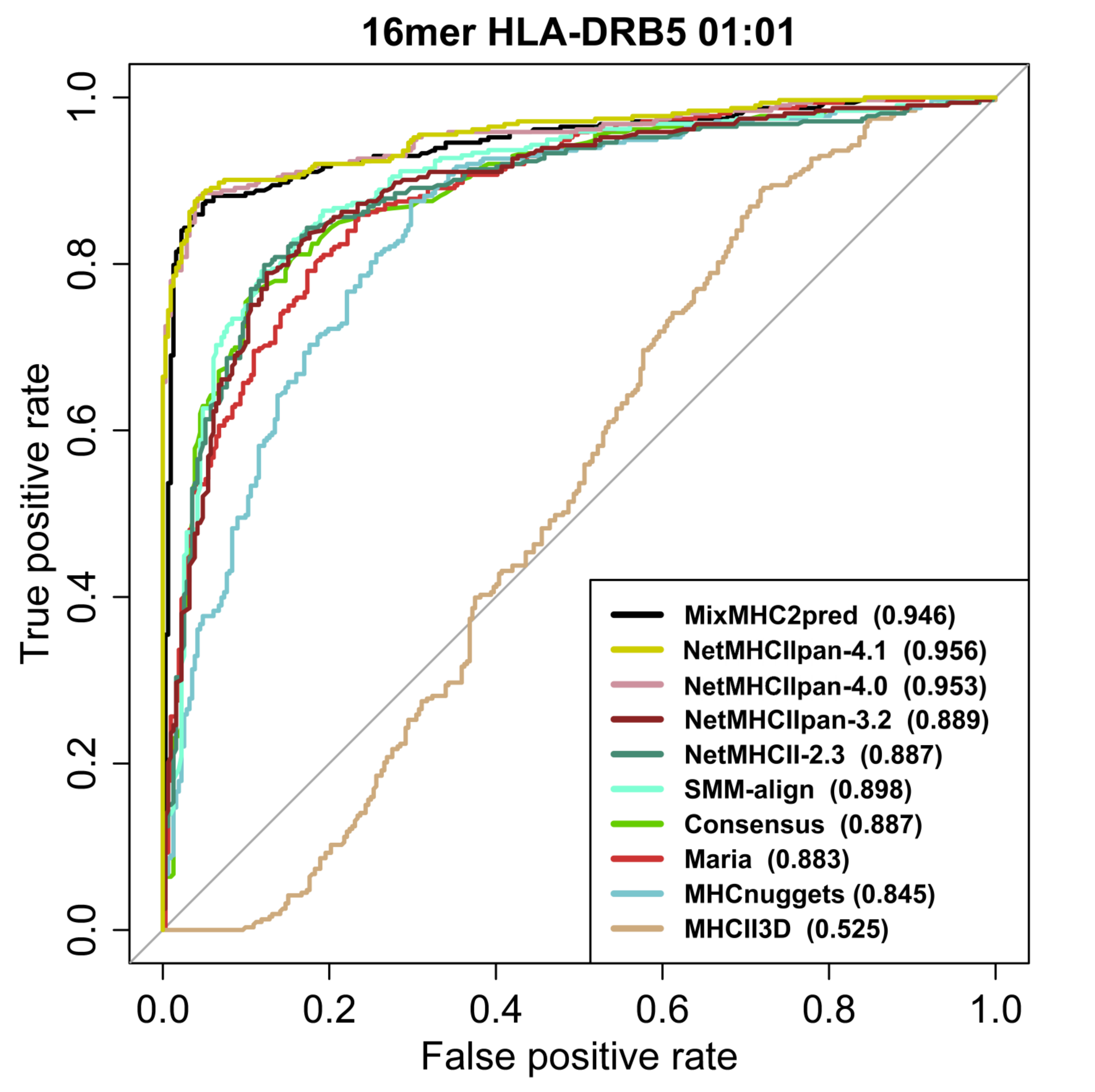

16mer HLA-DRB5 01:01
True positive rate
False positive rate
0.0
0.2
0.4
0.6
0.8
1.0
MixMHC2pred (0.946)
NetMHCIIpan-4.1 (0.956)
NetMHCIIpan-4.0 (0.953)
NetMHCIIpan-3.2 (0.889)
NetMHCII-2.3 (0.887)
SMM-align (0.898)
Consensus (0.887)
Maria (0.883)
MHCnuggets (0.845)
MHCII3D (0.525)
